# Implementation of evidence-based practice: The experience of nurses and midwives

**DOI:** 10.1371/journal.pone.0256600

**Published:** 2021-08-27

**Authors:** Asrat Hailu Dagne, Mekonnen Haile Beshah

**Affiliations:** Department of Midwifery, Debre Tabor University, Debre Tabor, Amhara Region, Ethiopia; Jordan University of Science and Technology, JORDAN

## Abstract

**Background:**

Implementation of evidence-based practice in clinical practice is crucial. Nurses and midwives play a vital role in using updated evidence. However, limited support and barriers to implementing evidence-based practice hamper the use of up-to-date evidence in clinical decision-making practice. Therefore, this study aimed to explore the implementation of evidence-based practice of nurses and midwives working in public hospitals.

**Methods:**

A qualitative descriptive study was conducted to explore the experience of implementing evidence-based practice among nurses and midwives working in public hospitals. A total of 86 participants, of which, 25 in-depth interviews, 5 FGDs having 47 participants and 14 participants were involved during observations, were considered in Amhara Region public hospitals from November 17, 2019 to April 25, 2020. The observational data, interview and FGD transcripts were imported into NVivo 12 plus to manage and analyze the data using the Computer-Assisted Data Analysis Software Program (CAQDAS). The data were analyzed through thematic content analysis.

**Results:**

Nurses and midwives perceived that implementation of evidence-based practice is the use of research findings, guidelines, hospital protocols, books, and expert experience in clinical decision-making practice. However, there was limited support for the implementation of evidence-based practice by nurses and midwives. The lack of knowledge and skill to use evidence like research findings, time mismanagement, the lack of motivation, the lack of resources and training were the perceived barriers to the implementation of evidence-based practice. Stick to the traditional practice due to lack of incentive and unclear job description between diploma and BSc nurses and midwives were the perceived causes of the lack of motivation.

**Conclusions:**

The experience of evidence-based practice of nurses and midwives indicated that there was limited support for the implementation of evidence-based practice. However, research findings were rarely used in clinical decision-making practice The Knowledge, attitude towards implementing evidence-based practice, lack of resources and training, time mismanagement and lack of motivation were the barriers to the implementation of evidence-based practice. Therefore, the promotion of adopting the implementation of evidence-based practice and training on the identified barriers are mandatory.

## Background

Implementation of evidence-based practice (IEBP) refers to the use of best, valid, currently available and relevant research findings, expert opinion, standard guidelines and books in clinical decision-making practice [1]. IEBP improved quality healthcare and client outcomes in the care setting like reducing patient pain, hospital stay and ulcers due to pressure [2]. Therefore, future research needs to explore ways to foster the documentation of evidence-based practice (EBP) interventions more effectively. Nurses and midwives who have higher educational status, and management and service provision experience can reduce barriers to the IEBP. Thus, IEBP achieves quality health care through knowledge, skill, the experience of health service providers, collaborative decision making and good time management [3].

The best, valid, currently available, and relevant research findings were rarely used in healthcare and clinical decision-making practice [4]. Nurses and midwives use experienced-based knowledge, and their observations, colleague and other collaborators’ support in practice without considering best and current evidence [4]. International and national organizations have enhanced IEBP for the standard of quality health care service. IEBP is essential to meet patient safety and quality health services. It is also vital to increase formal and informal health information, treatment expectation, and patient role related to clinical decision-making practice [5]. Several standard guidelines, books, primary research and systematic review results are produced continually [6].

The uptake of EBP by updating knowledge, skill and attitude of nurses and midwives improved the advanced practice of nurses and midwives through role modeling, training, problem-solving and facilitating change [7]. However, nurses’ and midwives’ education for master’s and Ph.D. holders is not common even in European countries like France to implement evidence-based in clinical and healthcare practice, and research is conducted and is well known to use it for clinical decision-making practice in higher educational institutions [8]. Worldwide, the quality of research and standard guidelines engaging in evidence-based behavior is low. In addition to this, most factors influencing IEBP are not well identified and there is a call for further research to be done globally [9]. The study conducted in South Africa indicates that the use of evidence like research, standard guidelines and books require time and perseverance from international researchers and stakeholders together with a readiness by local researchers and stakeholders to take and actively promote IEBP in clinical and healthcare practice [10].

IEBP involves solving complex problems that are basic in healthcare [11]. Nurses and midwives have to address IEBP gaps through the insertion of the evidence into clinical practice, i.e., research findings, currently updated experts opinions, standard guidelines, and books. To fulfill this proposed role, they have to prepare their clinical expertise [12]. Studies suggested that IEBP is intervened by an interplay between the individuals, the new knowledge, and the actual context in which the sources of evidence are to be organized and utilized in daily practice [13, 14]. In addition to this, IEBP should be locally evaluated and the evaluation results must be made actionable and usable, and adapted to the local situations to get the best-needed outcomes [15–17].

There is a paucity of literature that explores nurses’ and midwives’ experiences towards barriers and supporting factors of IEBP in Ethiopia. The study’s findings will serve as a baseline for measuring and monitoring change in IEBP readiness following a tailored educational and organizational intervention. Therefore, this study was designed to explore the IEBP of nurses and midwives, barriers and supporting factors that affect the implementation of evidence-based practice among nurses and midwives.

## Methods

### Study design and setting

A qualitative descriptive study was engaged from November 17, 2019 to April 25, 2020 to explore the experience of IEDBP among nurses and midwives working in Amhara Region Public Hospitals. Three specialized hospitals (Debre Birhan, FelegeHiwot, and Gondar), four general hospitals (MehalMeda, Motta, Debark, and FinoteSelam) and eighteen primary hospitals (Dembia, Durbetie, Deneba, Debre Sina, ShoaRobit, Feres Bet, Ataye, Adet, Addis Alem, MekaneEyesus, Addis Zemen, Merawi, Burie, Wogera, Delgi, Nefas Mewcha, Wogeda, andMetema) were involved in the study.

### Participants, sampling and recruitment

A total of 86 participants were considered for the in-depth interview (8 key informants and 17 interviewees), five FGDs (47 participants) and fourteen observations were conducted. The key informants included three hospital managers, two medical directors and three case managers (masters in emergency surgery and obstetrics). Each focus group discussion (FGD) consisted of eight to twelve participants. A checklist was employed to observe nurses’ and midwives’ roles, and the availability of resources/materials used for EBP in the fourteen hospitals. Nurses, midwives, doctors and masters in the emergency surgeon and obstetric participated in the in-depth interview. Nurses and midwives participated in FGDs. Name of nurses, midwives, doctors and masters in the emergency surgeon and obstetric was coded for the participants of the interview (101–125), FGD1 (FGD1-01 to 9), FGD2 (FGD2-01 to 10), FGD3 (FGD3-01 to 10), FGD4 (FGD4-01 to 08), FGD5 (FGD5-01 to 10) and observation (H101- H114) (see S1 File). A purposeful sampling strategy was used to select hospital managers, medical directors and case managers who have leadership roles as key informants. A similar strategy was used to select nurses and midwives for participation in the interview, FGD and observation. Participants who give optimal insight into the implementation of evidence-based practice were recruited through the hospital managers and the heads of department, and their contact details were obtained. Due to the busy schedule of participants, a pragmatic approach was favored to get them for the interview and FGD. A calendar invitation was subsequently sent out inviting the professionals to participate, and all agreed.

### Data collection

The theoretical framework of Klein and Knight was used as the basis of the FGD and interview guide [18]. The in-depth interview and FGD guides and checklist for observation were developed by reviewing literature and feedback of experts in IEBP. The theoretical framework consisted of factors enhancing and challenging the IEBP. IEBPs established by an organization, shared perceptions of the IEBP, a supportive organization to IEBP, the availability of resources and a learning organizational knowledge and skill development were enhancing factors. However, unreliable evidence, lack of knowledge and skill, time mismanagement, lack of motivation, the decision to adopt and implement an innovation made by higher up in the hierarchy than the innovation’s target users and lack of commitment are the challenges of implementation of evidence-based practice.

The interview and FGD guide were divided into four sections i.e. Socio-demographic variables, perceptions towards IEBP, perceived barriers of IEBP and supporting factors for IEBP. The in-depth interview and FGD guide were also prepared first in English then translated to Amharic and retranslated back to English for consistency. Eight data collectors (research assistance) who had the educational status of master and Ph.D. with previous qualitative data collection experience were selected. They were trained to be familiar with the objective and the methodology of the research. Data were collected via FGD and face-to-face interview techniques using semi-structured questionnaires. Good communication started with the greeting and the ground rule had been set before the focus group discussion started. The interviews and FGDs took place in separate rooms at hospitals that guarantee good communication. The interview duration was between 45 minutes and 60 minutes and the FGD duration was 90 minutes to 120 minutes. Moreover, the data collectors were engaged in participatory observation using a checklist. The participants’ emotions and non-verbal communication were recorded as field notes. The interviews and FGDs were audio-recorded and then later transcribed for analysis. Saturation was determined when there were multiple overlapping responses across participants.

### Data analysis

Interview and FGDs data were captured using voice recorders, and each day field notes were transcribed verbatim first in Amharic and then translated into English and retranslated back to Amharic by interviewers and FGD data collectors each day to check for consistency. The transcripts were read repeatedly and checked independently by investigators for confirmation. Initially, the observational data, and interview and FGD transcripts were imported into NVivo 12 plus to manage and analyze the data using the Computer-Assisted Data Analysis Software Program (CAQDAS). The data were analyzed through content analysis. First, a list of codes was created and described. Then after adding and defining the concept, categories were developed. The number of categories was reduced by” collapsing those that are similar or dissimilar into broader higher-order categories” [19]. Finally, the codes were ordered into essential categories, and the main contents and categories were identified. Moreover, essential quotations were clustered. The quotations were used to elaborate on the context that affects the participants’ experience and how the participants experienced the events.

### Trustworthiness

The investigators, research facilitators, nurses and midwives expert were invited to review the study’s findings and the right idea that represents their point of view was taken for the study to maintain credibility. Dependability was addressed by analyzing all the observational data, and interview and FGD transcripts by at least two researchers with a third “checker” to ensure consistency across the data analysis process. Moreover, the investigators and research facilitators discussed the emerging categories from the dataset and resolve any different perspectives by foraging consensus on interpretation. The decision of transferability of the findings to a new set of situations depend on the contextual information provided by the investigators.

### Ethics approval and consent to participate

Ethical approval was obtained from the Ethical Committee of Debre Tabor University, health Science College and we communicated it to Amhara Region Ethical Review Board. A formal letter of cooperation was written for Amhara Region Public Hospitals and permission to conduct the study was obtained from the hospital and the unit managers. Participants were informed that they had the right to withdraw from the study at any time. Moreover, we informed the purpose, procedures, advantages and disadvantages. Finally, informed written consent was obtained from each study participant.

## Results

The participant’s ages ranged from 21 to 50 years, and their mean age was 31 years. Thirty-eight participants were married and thirty of them were single. Sixty male and twenty-six female participants have participated in the study. The work experience of the participants ranged from 1 year to 34 years and its mean was seven years. Of the total 86 participants, 46(53.5%), 32(37.2%), 8 (9.3%) were nurses, midwives and key informants respectively. Five MSc nurses, forty-one BSc nurses, three MSc midwives and twenty-nine BSc midwives participated in the study. In terms of participants’ positions, six head nurses and three head midwives participated in the study. Two medical directors, three hospital managers, three quality health care cordinators, and three case managers also participated in the study.

The data analysis of observation, FGD, and interview produced four themes. These four themes were the perceptions towards implementing EBPe, the nurses’ and midwives’ attributes the supporting of nurses and midwives for the IEBP and the perceived barriers to implementing EBP. The four themes were further subdivided into eleven subthemes. Of which, four subthemes were included under the nurses’ and midwives’ attributes, three subthemes were included under the supporting of nurses and midwives for IEBP and four subthemes were included under the perceived barriers to implementing EBP.

### The perceptions towards the IEBP

This is the theme defined as the awareness of participants towards the IEBP. The interviewees and FGD participants perceived that IEBP is using research findings, guidelines, hospital protocols, books, and experts’ experience during health services, particularly in clinical decision-making. One of the interviewees describes the perception towards the IEBPe like this:

“*First of all, evidence-based practice is the use of hospital protocols, guidelines and training manual for health care service especially when we give patient care and do procedures. It is a matter of reading books and search for research findings. It is also to get updated information during morning sessions and seminar presentations from experts’ experience (119)*.”

Another focused group discussion participant expressed his perception as follows:

“*I understand that EBP is the use of scientifically proved evidence in the health service. It is a means of clinical practice based on rules and follows the scientific procedure (FGD5-02)*.”

### The nurses’ and midwives’ attributes

The nurses’ and midwives’ attributes are personal attributes that affect the ability to implement EBP. The nurses’ and midwives’ attributes include knowledge, skill, attitude and experience in IEBP. Knowledge, skill and attitude indicate the credibility of nurses’ and midwives’ expertise in implementing EBP. However, nurses and midwives felt that they did not have the knowledge, the skill and the attitude to implement EBP. Further, they could not differentiate quality research. This was described by one of the interviewees:

“*I am working in all wards and my colleagues too…*. *I do not expect knowledge and skill at the competency level to use evidence like standard guidelines*, *books and research findings*. *I do not think that we have the knowledge and the skill to perform every procedure using evidence particularly quality research*. *Some of the nurses and midwives may not be positive to read the evidence*. *They are negligent in using evidence (121)*.”

The observation of BSc midwife using a checklist revealed that there were challenges to perform tasks without difficulty. Skilled delivery was attended without the steps of procedures. The skill and getting ready to perform the procedure were the practical challenges (H111).

### Experience in IEBP

Experience in IEBP is the subcategory of nurses’ and midwives’ attributes. It is participants’ experience in using evidence such as standard guidelines, hospital protocols, experts’ opinions, books and research findings. Nurses and midwives use guidelines and hospital protocol for their day-to-day activities. Sometimes, they read books to increase their confidence in clinical decision-making practice. However, they use research findings to provide health services rarely. The experience of nurses and midwives in IEBP was stated by one of the FGD participants:

**“***You see*, *implementation of evidence-based practice is beneficial*. *Medicine is updated every time*. *Evidence that we use today may not work for tomorrow*. *Most of the time*, *I use guidelines and hospital protocols*. *However*, *I didn’t use a research article*. *Even if*, *there is an off library in our hospital*, *we refer to some books*. *We know our job is teamwork and there is supporting and sharing ideas between team members* (FGD4-03).”

### The supporting of nurses and midwives for IEBP

The theme ‘the supporting of nurses and midwives for IEBP’ included the subthemes ‘the supportive organization to implement EBP’, ‘the support from Non-governmental Organizations (NGOs) and other stakeholders to implement EBP’ and ‘the supportive supervision, monitoring and evaluation of IEBP’. The support for nurses and midwives to implement evidence-based practice and managers’ role in supporting organizations affect the ability to implement EBP.

### Supportive organization to implement EBP

Nurses and midwives’ managers and ward heads experienced that they did not support organizations to implement EBP. However, they understand that supportive organizational resources like electronic journals, work-based libraries, books and research findings had an impact upon IEBP. This was described by one of the interviewees:

“*I ask chief manager, “how to implement evidence- based practice through training and fulfill resources in the hospital?” I did nothing beyond this. I cannot communicate with higher officials and other stakeholders outside this hospital. The chief manager can do this. I understand using guidelines, books, hospital protocols, training manuals and experts’ opinions to improve quality health care. (124)*.”

There should be a strong supportive organization for the existence of the IEBP. However, nurses and midwives felt no support and commitment in using updated guidelines, journals and protocols. This was described by one of the FGD participants:

“*Updated evidence like guidelines, hospital protocols, books and journals should be available to apply the evidence-based practice. I do not think that we are using updated guidelines and hospital protocols. As a nurse and midwife, commitment is essential. We have to support each other and get support. I do not remember this was done practically (FGD3-06)*.”

### NGOs’ and other stakeholders’ support for IEBP

One cannot expect the IEBP without the support of both local and international stakeholders. Non-governmental organizations like the world health organization, the health bureau and the Ethiopian ministry of health support hospitals by providing training for nurses and midwives, distribution of guidelines and training manuals. However, the NGOs’ and other stakeholders’ support for implementing EBP is still inadequate. There is no training considering research findings to use in clinical decision-making practice and healthcare. One of the labor and delivery ward head midwife describe the stakeholders’ support as follow:

“*There are NGOs like CDC*, *World Vision and Gender Health Ethiopia that provide short-term training and different guidelines*. *These organizations did well but it is not adequate support*. *We use these updated guidelines*. *Nevertheless*, *there is a big gap in using research articles and there are no supporters to use it (105)*.”

### The supportive supervision, monitoring and evaluation of IEBP

Mentoring and supportive supervision, control and evaluation were identified in all FGD and interview participants to change IEBP. One of the interviewees stated his experience:

”*What do you mean*? *How could we change without the mentoring and supervision of the IEBP*? *I understand that it is important*. *However*, *there is no direct mentoring and supportive supervision*, *control and evaluation of nurses’ and midwives’ use of guidelines*, *books and manuals*. *We do it indirectly*. *I do not expect something good towards the use of research in our clinical settings (112)*.”

### The perceived barriers to IEBP

The theme ‘the perceived barriers to IEBP’ involved the subthemes ‘knowledge and skill’, ‘insufficient time’, ‘lack of resource and training’, and ‘lack of motivation’. The data analysis of interview, FGD, field note and observation identified the barriers of IEBP.

### Insufficient time

All the study participants described that shortage of time was a barrier to implement EBP. One of the emergency ward head nurses described this barrier as follow:

“*We do not have any plan of implementation of evidence-based practice because of emergency activities*. *We work for 24 hours*. *We are too busy*. *We can get different pieces of evidence from the library*. *However*, *we cannot go to the library due to a lack of time in this emergency ward*. *Nurses are few compared to emergency activities*. *Nurses are working continuously*. *They are strong*. *Nurses do not use evidence because of the workload*. *As I told you*, *we have a shortage of time*. *I have no time to read books*. *There is tiredness*. *We sleep*, *when we get time*. *We cannot consider anything rather than this because of a shortage of time (111)*.”

### Lack of resources and training

The data from FGD, interview, field note and observation indicated that participants did not implement EBP because of a lack of guidelines, hospital protocols, books, research articles and training. One of the key informants described the condition:

“*Nurses and midwives use guidelines, books and hospital protocols. However, it is not easy to get books, guidelines and recent research. Nurses and midwives do not understand the best research findings. They do not have the skill to use it and we need training. We cannot get updated guidelines and manuals for most of the procedures (122)*.”

During the hospital visit, the observation revealed that most of the procedures did not have guidelines and hospital protocols and there were no books and literature in the ward. Moreover, most of the hospitals had no library, computers and internet access in the wards. The nurse claimed the nonuse of these resources (H105).

### Lack of motivation

The majority of interviewees and FGD participants believed that motivation is one of the driving forces of implementing EBP. They describe personal derive and motivation to change the IEBP. It is impossible to change the existing EBP of health care and clinical decision-making without personal drive and motivation. This was described by one of the key informants:

”*Let me tell you the real history of an educated patient and doctor*. *The doctor has been a long time in the hospital*. *He was frustrated and he was as he had been graduated from college*. *There was no incentive for him to do his job and negligence is his habit which does not lead to a good attitude*. *The patient knows the doctor very well*. *The patient got this doctor during the examination*, *and the patient said that no…he did not read anything about his profession after he had been graduated*. *The patient went to another doctor who was working in the university thinking that he read many books and articles*. *Similarly*, *nurses and midwives were not motivated to update themselves through reading books*, *guidelines and research articles*. *They stick to traditional practice (108)*.”

Nurses and midwives blamed that having unclear job descriptions decrease their interest and motivation in doing their job. One of the participants stated that an unclear job description decreases motivation to implement EBP.

“*There are no job descriptions of MSc, BSc and diploma nurses and midwives. There is no clear demarcation of job descriptions among nurses and midwives based on the level of education. You see here, this is a matter of morals. I feel less interested when I am always doing the same job of diploma (101)*.”

## Discussion

This study presented that the participants’ perception towards IEBP, the nurses’ and midwives’ attributes, the supporting of nurses and midwives for IEBP, and the perceived barriers of IEBP were the main themes of analysis. The participants had an understanding of how to implement EBP. The supporting of nurses and midwives to implement EBP indicates quality healthcare and working for safe patient care [2, 20]. Barriers to IEBP of nurses and midwives were the primary concern in the experience of IEBP [21, 22].

In this study, participants were seen to perceive the concept of IEBP. They understood that IEBP was using different types of evidence as a source of knowledge and skill in clinical decision-making practice and healthcare service. They know which types of evidence they used frequently. Commonly, participants use guidelines, hospital protocols and training manuals for clinical decision-making practice in our study conducted among nurses and midwives working in Amhara Region public hospitals. The studies conducted in Ghana and England were in line with this finding which indicated participants’ understanding of IEBP [7, 20]. However, our study findings showed that research was rarely used. This is because of a lack of understanding to use research and participants’ trust to use it for IEBP.

This study conducted in Amhara region public hospitals revealed that there was little support for nurses and midwives to implement EBP, and managers’ and ward heads’ commitment to support nurses’ and midwives’ IEBP was not successful. Moreover, nurses and midwives use guidelines and protocols without concern for the update. This finding let us understand the support for IEBP consisted of all the management team that provided mentorship, delivering resources and commitment to collaborative activities. This finding agrees with the studies that reported that barriers and facilitators to EBP occurred at the organization and individual level [21–24]. The reason could be less support of staff to decrease barriers of IEBP due to lack of managers’ commitment, poor access of resources and lack of expertise to share updated information in all studies with the same finding. Therefore, there should be support where there were barriers to the IEBP.

This study identified that the barriers to implementing EBP were time mismanagement, lack of knowledge, negative attitude, lack of motivation, lack of resources and training. These barriers could be categorized under individual and institution level barriers. Studies conducted in Canada, Ghana, Germany, Iran, China and Jordan presented these barriers to implementing EBP [20, 22, 23, 25–27]. However, the perceived causes of these barriers to use guidelines, hospital protocols, books, experts’ opinions and research in our study were different from causes of barriers to IEBP in other studies. In our study, participants perceived that barriers to implementing EBP stem from the lack of incentive and unclear job descriptions between diploma and MSc nurses and midwives. Diploma and MSc nurses and midwives were seen to have the same clinical practice activities because of unclear job descriptions between diploma and MSc nurses and midwives. Participants had no interest in implementing EBP. Our study findings also indicated that nurses and midwives did not read books, guidelines and research in clinical decision-making practice due to negligence in doing the same thing. Otherwise, the USA’s study revealed that the lack of motivation was common among nurses working for long years in one health facility which resulted in the loss of interest due to the length of time between formal academic training and current employment [28].

Denmark’s study also revealed that lack of motivation of nurses presented and the perceived cause of lack of motivation was failed IEBP due to nobody taking action on the agreed plan [29]. Otherwise, our study also presented that the participants were inclined to traditional clinical practice. Our study participants think that the use of updated evidence added the burden or workload to their day-to-date activities. They were negligent and they were not interested. They were not motivated. They also wanted incentives and support to implement evidence-based practice.

Our study revealed a lack of supportive organizational resources to get electronic journals, a work-based library, and access to books and research. Otherwise, the lack of support presented in the study of Denmark was non-formalized at the organizational level. Denmark’s study had no problem of sharing new sources of evidence and consensus decision to use the new evidence [29]. The possible reason for this difference in this lack of support for both studies could be better promotion of implementing EBP in Denmark.

One of the outcomes identified as subthemes was “the lack of resources and training.” The participants had difficulty of getting updated guidelines, books and research findings. They had a knowledge gap even if they got journals and they wanted training to use research. As far as our search for other similar studies, no other studies reported this finding.

### The strengths and limitations

The study’s major strength is the use of interviews, FGDs and observation data collection techniques that contributed to providing insight into the complexity of IEBP in the health care system. The risk of bias was restricted by ensuring privacy for the interviewee and a quiet room to conduct FGD. This study addresses that IEBP in this study is typically underpinned through addressing supporting factors for IEBP, perceived barriers of IEBP and the experience IEBP.

The first limitation of this study was the possibility for social desirability bias as the study was conducted using interview and FGD methods, while nurses and midwives were working in the hospitals. Moreover, the response of the participants might be inflated or underestimated due to individuals with some interests. Second, this study was conducted in hospitals where a more advanced human resource dynamic, quality medical service and well-organized structure were available. Hence, transferability is difficult for health centers and health posts.

## Conclusions

The experience of IEBP indicated that there was limited support for IEBP. Nurses and midwives used guidelines, hospital protocol and training manuals in the clinical decision-making practice. However, the research was rarely used in clinical practice. The knowledge, attitude towards IEBP, lack of training, time mismanagement and lack of motivation were the barriers to implementing EBP. The study’s findings will serve as a baseline for measuring and monitoring change in IEBP readiness following a tailored educational and organizational intervention. To implement EBP and to provide high-quality healthcare, organizational and individual level support for the IEBP is crucial. Moreover, the promotion of adopting EBP and training on the identified barriers are mandatory. Future research should be conducted to see the impact of IEBP on the quality health care.

## Supporting information

S1 File Availability of data and materials. The data set necessary to replicate our study findings as supporting information files.
(DOCX)

S2 File Tools. The interview, FGD and observation guides used in the study were both Amharic and English language.
(DOCX)
