# Supplementary material for: Implementation of evidence-based practice: The experience of nurses and midwives
Source: PLoS One. 2021 Aug 27;16(8):e0256600. doi: 10.1371/journal.pone.0256600 (PMC8396772; doi:10.1371/journal.pone.0256600)
Supplement: S1 File — The data set necessary to replicate our study findings as supporting information files. (DOCX) [file pone.0256600.s001.docx]

**Transcribed and translated data from FGD and interview, and observational data of the study conducted in Amhara Regional Sate of public hospitals**

FGD1

1. **How do you perceive the implementation of Evidence-based Practice in your hospital? How do you understand the implementation of evidence-based practice?**

The response of participant: FGD1-03

I know the implementation of evidence-based practice. For example, I know malaria distribution from research. I also use my colleagues as a source of knowledge and skills.

The response of participant: FGD1-06

I know the implementation of evidence-based practice. It means showing the direction of activities done during healthcare clinical practice. I use guidelines and hospital protocol for this. The use of these guidelines and hospital protocol is the implementation of evidence-based practice.

1. **How do you think about the importance of the implementation of evidence-based practice? What are the sources of evidence used for the implementation of evidence-based practice?**

The response of participant: FGD1-02

I know there are many sources of evidence. For example, knowledge or information about malaria patients can be obtained from research to use as a source of evidence. Internet is the source of evidence. I can get updated information from the internet. I know that I can get some procedures directly from the internet. I also know that I can get books and guidelines from the internet.

I implement evidence-based practice through reading national guidelines and hospital protocols. In addition to this, I read new updated information from internet searching. I use my colleagues as a source of evidence.

The response of participant: FGD1-05

I use guidelines and hospital protocols to order drugs and laboratory requests. I use sources of evidence that I got during school education. I read updated books to implement evidence-based practice. In addition to this; I use research findings for my implementation of evidence-based practice.

The response of participant: FGD1-07

I know that the implementation of evidence-based practice has good outcomes for the patient. When I see the intervention of research-based studies of malaria, it has good outcomes. I can get knowledge and skills from magazines, the internet and television. I use the hospital protocols. I read these sources of evidence every two days and sometimes. I read it even every month.

1. **How do you think your / nurses’ and midwives’/ knowledge, skills, and attitude towards the implementation of evidence-based practice?**

The response of participant: FGD1-09

Our handling of sources of evidence and implementation of evidence-based practice is low. There is a gap in skills and awareness. Providing training for nurses and midwives is important to fulfill these gaps to implement evidence-based practice.

1. **Why don’t you implement evidence-based practice?**

The response of participant: FGD1-03

There are many barriers to implement evidence-based practice. Work overload prevents nurses’ and midwives’ implementation of evidence-based practice. The other problem is administration issues like loss of freedom through the frustration of nurses and midwives by abusing them using attendance follow-up to influence them. The other barrier is a shortage of logistics like pens and notebooks.

The response of participant: FGD1-07

I do not implement evidence-based practice because of workload and shortage of internet access. I am not satisfied with my job. The hospital should fulfill internet access, library and other newly updated sources of evidence like guidelines.

The response of participant: FGD1-08

I do not implement evidence-based practice because of the lack of availability of guidelines and hospital protocols.

The response of participant: FGD1-09

I do not implement evidence-based practice because of less interest and commitment. The other reason is the availability of sources of evidence in the hospital. For example, there is no internet access, hospital protocols and other sources of evidence. Lack of perception and attitude is another problem.

The hospital should fulfill the sources of knowledge and skills like books and updated guidelines for nurses and midwives. These sources of evidence should be placed everywhere for nurses and midwives. Workload hinders the use of these sources of evidence. Workload causes tiredness and shortage of time.

There is no internet access in our hospital. I use hard copies of guidelines for the implementation of evidence-based practice but there are no updated guidelines.

In our hospital, many things should be fulfilled and it is very simple. I tried to implement evidence-based practice. However, there is a gap in the hospital to fulfill sources of knowledge and skills.

1. **How the supports for the implementation of evidence-based practice important?**

The response of participant: FGD1-01

I know that there is managers’ support regarding the implementation of evidence-based practice. However, there are different challenges to manager’s support. For example, salary improvement and incentives are beyond manager’s activities.

The response of participant: FGD1-05

I have never seen managerial support for the implementation of evidence-based practice in our hospital. The manager and heads simply follow which activity is well done and which one is not. They do not know what is important for the implementation of evidence-based practice during healthcare and clinical decision. There is no way to address the implementation of evidence-based practice". Shown his angry face.

The response of participant: FGD1-06

I know that managers and heads do not supervise and control our implementation of evidence-based practice. They follow about patient care without the best evidence-based practice. They focus on who provides good care for the patient. They give a result based on outcome-based services. However, we do not know about the implementation of evidence-based practice.

The response of participant: FGD1-09

Managers should supervise and control the implementation of evidence-based practice among nurses and midwives. However, our managers and ward heads do balanced score cards. They do not encourage the implementation of evidence-based practice. There should be staff discussion on the implementation of evidence-based practice. Managers should fulfill resources and access for the implementation of evidence-based practice.

The response of participant: FGD1-05

Keeping nurses’ and midwives’ morale through increasing salary and incentives encourage the implementation of evidence-based practice. I read books to implement evidence-based practice. Reading needs morale and motivation. First of all nurses and midwives should get adequate payment for what they are performing. Implementation of evidence-based practice is tedious. It needs incentives.

The response of participant: FGD1-06

Evidence used for the clinical decisions should be available. For example, drug information center should be established. Internet access should be available in all wards. Our commitment is important despite the access to books, guidelines and national protocols. Managers and heads should introduce the importance of implementation of evidence-based practice through training and discussion during the staff meetings. Managers and heads supervise and control nurses and midwives’ implementation of evidence-based practice.

The response of participant: FGD1-03

Implementation of evidence-based practice improved through giving a reward for nurses and midwives. In addition to this, encouraging the role models of nurses and midwives increase the implementation of evidence-based practice.

FGD2

1. **How do you perceive the implementation of Evidence-based Practice in your hospital? How do you understand the implementation of evidence-based practice?**

The response of participant: FGD2-01

I understand that the implementation of evidence-based practice solves the patient problems because we observed better outcomes for the patient when health professionals implement evidence-based practice.

The response of participant: FGD2-03

I understand that health professionals cannot provide health services without the implementation of evidence-based practice.

The response of participant: FGD2-10

I know the time is a revolution for the implementations of evidence-based practice. I understand we implement evidence-based practice.

The response of participant: FGD2-07

I have perception and knowledge about guidelines’ use as sources of knowledge and skills. We

do not read research articles in healthcare and clinical practice. Our hospital staff uses guidelines for the implementation of evidence-based practice. However, the senior doctors tell us about research results to use as a source of knowledge during clinical decision-making practice.

1. **How do you think about the importance of the implementation of evidence-based practice? What are the sources of evidence used for the implementation of evidence-based practice?**

The response of participant: FGD2-01

Let me tell you about the best evidence-based practice. I see one study in America that indicates prehypertension (130/80 mmHg) in our diagnosis is diagnosed as hypertension in America. We can get such sources of evidence from research articles. Our university is not familiar but I have known there is annual research conference participation in Gondar University. These important research reports were from the operation room side, anesthesia, midwives, nurses and others. There were seniors and professors during the report presentation. These articles were published and available in the library for health professionals to use it. When I come to our situation, there are not researchers. There is not access to research articles for the implementation of evidence-based practice. When I get access to sources of knowledge and skills. I use it for my implementation of evidence-based practice.

The response of participant: FGD2-02

Implementation of evidence-based practice has many uses. It is used for better accountability and identifies patient care. Implementation of evidence-based practice is important. It is used for better accountability and identifies patient care.

The response of participant: FGD2-03

Use of research articles should be included in hospital protocols. Doctors and other health professionals can use it without fear. There is a challenge to use research findings in healthcare and clinical practice.

The response of participant: FGD2-04

I can say that the implementation of evidence-based practice frees us from accountability when there is patient harm and it is useful for the patient.

The response of participant: FGD2-05

Implementation of evidence-based practice reduces patient harm, increases professionals’ knowledge and patient satisfaction.

The response of participant: FGD2-06

Implementation of evidence-based practice improves quality care service and patient satisfaction.

The response of participant: FGD2-07

I know the implementation of evidence-based practice improves patient harm and accountability.

It also increases patient satisfaction and helps for the achievement of the objectives of our hospital.

The response of participant: FGD2-08

Implementations of evidence-based practice determine quality care for the patient and patient satisfaction.

The response of participant: FGD2-02

I know national guidelines and other sources of evidence are used for the implementation of evidence-based practice. Study results are base for the implementation of evidence-based practice.

There are different guidelines that are updated every 5 to 6 years. These guidelines are available in the hospital’s library. There are also articles and procedure guides in the library. Health professionals download sources of evidence from the internet. I use only the hard copy of the guidelines

The response of participant: FGD2-03

There are different guidelines, research articles, and hospital protocols used for the implementation of evidence-based practice. There are hospital policies. What is included in the policies? What is included in the guidelines? One should provide health services based on these points to implement evidence-based practice. I see that most staff members do not use updated guidelines despite using textbooks and school knowledge. This is also a probability. I implement evidence-based practice using my previous knowledge and skills.

Our ward uses treatment guidelines for the implementation of evidence-based practice. The other evidence is drug formulary. We get these guidelines from the health bureau. The guidelines are note updated every 5 years. The third source of knowledge and skills is senior health professionals.

The response of participant: FGD2-04

Scientific sources of evidence, guidelines and hospital protocols are used for the implementation of evidence-based practice.

The response of participant: FGD2-05

There are training manuals and guidelines in our ward that we use for the implementation of evidence-based practice. In addition to this, trained staffs share ideas about the new training manuals so that we can use the new manuals for the implementation of evidence-based practice. The doctors also share their idea and we use them for the implementation of evidence-based practice.

The response of participant: FGD2-06

We use guidelines and training manuals like basic emergency obstetric and newborn care (BEONC) for the implantation of evidence-based practice. Research study results also important for the clinical decision but we do not use it.

The response of participant: FGD2-10

Most of the time, we use national guidelines and hospital protocols for our clinical decision. In addition to this, school education and research articles are useful for the implementation of evidence-based practice.

The response of participant: FGD2-09

Implementation of evidence-based practice is a professional obligation. I get the best sources of evidence from experienced health professionals. I get sources of evidence from the health bureau and the ministry of health. We can also get research articles when we are interested but we do not use it for clinical decisions. I use guidelines prepared by seniors.

The response of participant: FGD2-03

You see, the implementation of evidence-based practice is very useful. Medicine is updated every time. Evidence that we use today may not work for tomorrow. Most of the time, I use guidelines and hospital protocols. . Even if, there is an on and off library in our hospital, we refer to some books. We know our job is teamwork, there is supporting and sharing of ideas between team members. Regarding research article, I did not use it.

The response of participant: FGD2-08

W e can get evidence from different ways. There is a recommendation to use research evidence. For example, if caesarean section is done for a high number HIV viral load cases, it decreases mother-to-child HIV transmission. This is the evidence that we can get from research. I use these sources of knowledge from research. This information is useful for the patient.

The response of participant: FGD2-10

There are research articles from the internet. I use this for my knowledge but not for clinical decisions. Even though there is not training, we can download research articles, health policies and national guidelines and use these sources of evidence for clinical decisions.

1. **How do you think your / nurses’ and midwives’/ knowledge, skills, and attitude towards the implementation of evidence-based practice?**

The response of participant: FGD2-08

I think we have the knowledge and perception of evidence-based practice but we do not update ourselves for the latest research findings, books, guidelines and national protocols.

1. **Why don’t you implement evidence-based practice?**

The response of participant: FGD2-01

There are barriers to the implementation of evidence-based practice. There is a lack of availability of research articles, books, guidelines and training manuals. There are no researchers in a health institution.

The response of participant: FGD2-02

There are barriers to the implementation of evidence-based practice. These are lack of access to guidelines, the internet, and other sources of evidence. I do not use the internet because it harms my eye. The other barrier is the hospital library is far from the ward moreover it has no space and tables for reading materials in the library. Many people cannot use the library at the same time. Experienced colleagues are limited to their ward.

The response of participant: FGD2-03

We do not give attention to evidence-based practice. The other problem is increasing patient flow which causes a lack of time to read books, guidelines, research findings, etc.

The response of participant: FGD2-04

Some factors influence health professionals to implement evidence-based practice. These are an imbalance of health service providers and patient flow (workload), lack of internet access and limited access to sources of evidence used for the clinical decision to each ward.

The response of participant: FGD2-05

There are barriers to the implementation of evidence-based practices in our hospital. The guidelines and training manuals are present in different wards. I am in neonatal intensive care unit ward and I do not know the guidelines and manuals of the medical ward or other wards. Even I do not know the reason. The problem may be library or communication. Despite this problem, I use guidelines and manuals of my ward for the implementation of evidence-based practice.

Our hospital has not desktops for each ward as other hospitals have these. The desktops are in our manager or head office and we cannot get these computers. This prevents the staff to get sources of evidence from the internet for our implementation of evidence-based practice.

Participant response: FGD2-07

The factors that influence health service providers to implement evidence-based practice are lack of guidelines and internet access.

The response of participant: FGD2-08

When I see practical procedures, we do not follow the steps of procedures or protocol because of high patient flow. Our work is to give health care to all patients who come to our hospital.

The response of participant: FGD2-10

There is a lack of guidelines, internet access and standard books. The other is a shortage of time to use these materials and a lack of interest of nurses and midwives to read the sources of evidence. In my hospital, there is limited internet access. I believe that nurses and midwives do not use the internet during their free time. There is also a limitation to use the library. There are few books in the room of the library. These are not enough for nurses and midwives to implement evidence-based practice.

1. **How the supports for the implementation of evidence-based practice important?**

The response of participant: FGD2-02

Relating managerial support, there is monitoring and supervision. There is a quality care team that observes the daily, weekly and monthly activities of staff. The team encourages the best performance of the staff members and provides support for the poor performance of the staff members. Beyond this, managers say this activity is done, this is not done, you are not punctual and you are absent but they do nothing about guidelines and research articles for the implementation of evidence-based practice.

The response of participant: FGD2-06

Probably, non-governmental organizations supervise, control and support us to use training manuals, guidelines and protocols for our clinical decision. However, our managers and ward heads do not follow us on how to implement evidence-based practice.

The response of participant: FGD2-07

I see there is managerial supportive supervision in our ward. For example, there is a quality improvement team that follows a day-to-day activity of health service providers. The team has its project to give encouraging and supportive feedback.

The response of participant: FGD2-10

Mangers and head wards do not follow nurses and midwives whether they use guidelines, hospital protocol, research articles, or not. They do not have supportive supervision of the implementation of evidence-based practice. I have not seen any managers or heads who encourage the use of research articles and books.

The response of participant: FGD2-07

I do not know the use of research articles for the implementation of evidence-based practice.

FGD3

1. **How do you perceive the implementation of Evidence-based Practice in your hospital? How do you understand the implementation of evidence-based practice?**

The response of participant: FGD3-01

I know the implementation of evidence-based practice. It is a professional person’s knowledge and skills development through reading books and other sources of evidence and apply the information for his professional practice.

The response of participant: FGD3-02

I understand that it is the use of scientifically proved sources of evidence in the health service. It is a means of clinical practice based on rules and follows the scientific procedure to provide health care services for the clients.

The response of participant: FGD3-05

I know the implementation of evidence-based practice means the use of written documents obtained from universities, the internet and training.

The response of participant: FGD3-08

I know that the implementation of evidence-based practice is the use of standard evidence for clinical decisions during procedures.

1. **How do you think about the importance of the implementation of evidence-based practice? What are the sources of evidence used for the implementation of evidence-based practice?**

The response of participant: FGD3-01

The use of evidence-based practical minimizes mistakes, update knowledge and skills, to increase our knowledge, to get a good result and to improve accountability. In addition to this, evidence-based practice improves quality health care services.

The response of participant: FGD3-08

If we do our procedures based on hospital guidelines, we are free from accountability for patient harm. Implementation of evidence-based practice is useful for patient safety and quality health care.

The response of participant: FGD3-01

There are many sources of evidence used as sources of knowledge, skills and attitude. At the first level, one is expected to get his knowledge from college or University .At this level, the source of knowledge and skills is his teacher, books, the internet and guidelines.

I have not used books and read any research articles for my evidence-based practice. However, I use my colleagues and national guidelines as sources of knowledge and skills for my professional practice.

The response of participant: FGD3-02

For all disciplines, there are documented hard copies of guidelines. These are distributed to all wards so the professionals refer the evidence for clinical practice decisions. Moreover, referral books, articles and other sources of evidence to each discipline are available in all wards. When the professionals are not clear about their decision, they use these sources of evidence. I also use these sources of evidence sometimes, when I am not clear about my decision.

Most of the time, I use nursing standards for evidence-based practice. I use this standard for nursing diagnosis. Even if, there is an on and off library in our hospital, we refer to some books. It is possible to get the book by borrowing it from the hospital library. Regarding the research article, I did not use it. However, we know our job is teamwork, there is supporting and sharing of ideas between team members. I use health professionals for my evidence-based practice.

The response of participant: FGD3-03

We have updated national guidelines and world health organization guidelines. These are important for quality health care. I use mass media and my colleagues for the implantation of evidence-based practice.

The response of participant: FGD3-04

I use books, guidelines and my colleagues for my clinical decision during the implementation of evidence-based practice. We can get published sources of evidence like books and guidelines from the health bureau and our hospital in the form of hard copies. We use soft copies by sharing through our mobile. However, there is no access to soft copies. I can get research articles. Our colleagues share their knowledge with me during the morning session recently. Previously, we used 1 to 5 group discussion instead of the morning session.

The response of participant: FGD3-05

I select the best sources of evidence for my clinical decision to give quality care to the patients. I can get the sources of evidence from my colleague and organizations that give training for us. I can get guidelines, books and research articles from the internet.

1. **How do you think your / nurses’ and midwives’/ knowledge, skills, and attitude towards the implementation of evidence-based practice?**

The response of participant: FGD3-02

There are many sources of evidence used as sources of knowledge, skill and attitude. At first level, one is expected to get his knowledge from college or University .At this level; the sources of evidence are his teacher, books, and the internet. Guidelines are sources of knowledge and skills obtained from the internet. I feel that there is a gap of knowledge and skill to use the best sources of knowledge and skills.

1. **Why don’t you implement evidence-based practice?**

The response of participant: FGD3-01

Nurses and midwives have not got learning opportunities. We tried to improve our skills using sources of evidence. Moreover, implementation of evidence-based increases if there is a plan for a change of level of our education. Plan for change of the level of education encourages nurses and midwives to implement evidence-based practice. Because of a lack of learning opportunities, we are influenced to update ourselves using different sources of evidence.

The response of participant: FGD3-02

I see factors that influence evidence-based practice into two phases. The first one is the institution-related factors and the second one is the personal-related factors. Nurses and midwives work on other activities instead of their clinical practice. This is not the interest of nurses and midwives but the system of the hospital discourages them from their clinical decision practice.

We have a failure to read books, guidelines, protocols and articles. We do not identify our gaps and encourage good performance. There is a limitation of supporting weak staff members through training and encouraging competent staff members. These are factors that influence the implementation of evidence-based practice.

The response of participant: FGD3-06

I do not implement evidence-based practice because of my weakness. I always do the same procedure. Because of this, there is frustration. I feel what I can gain through reading. I think there is not development. We cannot apply our knowledge and skill that we get from college and university. There is a hierarchy that limits our knowledge and skills. For example, there is no job description among diploma, degree and master midwives. All do the same procedures. These conditions influence nurses’ and midwives’ implementation of evidence-based practice.

The response of participant: FGD3-07

Nurses and midwives do not implement evidence-based practice because of a lack of sources of evidence like internet access and guidelines, standards, manuals and so on. The other is the attitude problem of nurses and midwives. We have the idea of we know all things. No one knows more than me. Even when we do not know about one thing, we fear to ask others because of criticizes our knowledge. In this case, we do procedures without evidence.

The response of participant: FGD3-09

Relating implementation of evidence-based practice, the hospital has problems. It is not common to motivate nurses’ and midwives’ updating their knowledge and skills for the implementation of evidence-based practice.

There is no supportive supervision towards the use of updated sources of evidence for the implementation of evidence-based practice. In this hospital, there is a very great workload and I cannot read anything during working hours and at home. After working hours I go home and I sleep because of tiredness. The hospital has a shortage of books and guidelines to implement evidence-based practice. There is no internet access in this hospital.

1. **How the supports for the implementation of evidence-based practice important?**

The response of participant: FGD3-04

There is no hospital manager support to implement evidence-based practice. They are interested in taking attendance. They simply say who is late and absent. The managers do not have care about quality health care services. Their concern is how many of the patients get health service. Their concern is quantity rather than quality.

The response of participant: FGD3-06

Updated evidence like guidelines, hospital protocols, books, and journals should be available to apply the evidence-based practice. I do not think that we are using updated guidelines and hospital protocols. As a nurse and midwife, commitment is essential. We have to support each other and get support. I do not remember this was done practically.

The response of participant: FGD3-07

Managers’ support is very low to implement evidence-based practice. I see heads got national guidelines. It is better to discuss the guidelines instead of storing in their office. During 1 to 5 discussions, it is better to discuss evidence-based practice instead of politics.

FGD4

1**. How do you perceive the implementation of Evidence-based Practice in your hospital? How do you understand the implementation of evidence-based practice?**

The response of participant: FGD4-01

I understand that the implementation of evidence-based practice is important for the patient and health service providers. It has great value for quality health service. We perform our activities based on knowledge and skill obtained from different evidence in this hospital.

The response of participant: FGD4-02

Implementation of evidence-based practice means clinical and healthcare practice based on rules and follows scientific procedures to provide health care services for the clients.

The response of participant: FGD4-05

Implementation of evidence-based practice is to select the best evidence-based practice for healthcare and clinical practice to give quality care for the patients. I can get these sources of evidence from my colleague and organizations that give training for us. I can also get guidelines, books and research articles from the internet.

The response of participant: FGD4-06

I understand that the implementation of evidence-based practice is vital for the development of health service providers, improvement of quality health services and service provision based on ethics. However, there are different challenges

The response of participant: FGD4-08

**2. How do you think about the importance of the implementation of evidence-based practice? What are pieces of evidence used for the implementation of evidence-based practice?**

The response of participant: FGD4-02

I use outdated sources of evidence. Implementation of evidence-based practice is useful for the patient, hospital and health service providers.

The response of participant: FGD4-03

You see, the implementation of evidence-based practice is beneficial. Medicine is updated every time. Evidence that we use today may not work for tomorrow. Most of the time, I use guidelines and hospital protocols. However, I didn’t use a research article. Even if, there is an off library in our hospital, we refer to some books. We know our job is teamwork and there is supporting and sharing ideas between team members.

I do not implement evidence-based practice in clinical and healthcare practice. There are no updated sources of evidence in our setting.

The response of participant: FGD4-04

I know that the ministry of health distributed guidelines, national protocols and training manuals and we use it as a source of knowledge and skill. The hospital can use these sources of evidence after creating awareness for all health service providers.

The response of participant: FGD4-07

If we do our procedures based on hospital guidelines, we are free from accountability due to patient harm. Implementation of evidence-based practice is useful for patient safety and quality health care.

**3. How do you think your / nurses’ and midwives’/ knowledge, skills, and attitude towards the implementation of evidence-based practice?**

The response of participant: FGD4-04

I do have a concern about my knowledge and skill to use updated guidelines and national protocols. Most of us cannot use guidelines due to a lack of training.

The response of participant: FGD4-06

All things were mentioned. I have points that are not discussed. In our job, there is a need for updated knowledge and skills. We can update our knowledge and skills through training. Guidelines and training manuals are important for the implementation of evidence-based practice. In addition to this, we can use research articles and different books. Evidence-based practice is the use of sources of evidence based on principles and rules. For example, when nurses and midwives administer drugs, we have the knowledge that means we know the route of administration that is intramuscular, subcutaneous, and intravenous and others. We have also the skill. We know the benefit and the disadvantage of drugs. When we give treatment considering all the above things, we are implementing evidence-based practice.

**4. Why don’t you implement evidence-based practice?**

The response of participant: FGD4-01

I do not use guidelines in healthcare and clinical practice because of lack of updated guidelines, and national and hospital protocols, the problem of skills, lack of satisfaction, lack of motivation, lack of access to the internet, lack of reading room, work overload, lack of computer, lack of library and lack of skill lab demonstration.

The response of participant: FGD4-05

Nurses and midwives do not use guidelines, books, and national and hospital protocols in clinical and healthcare practice because of lack of library and reading room, work overload, lack of internet access, lack of computer in nurses and midwifery stations, lack of interest, lack of access to best and currently updated sources of knowledge and skills.

The response of participant: FGD4-06

Hospitals should find guidelines, books, protocols and research articles from different sites. There is not this kind of system. I am not quite sure of this communication. I think there is a break between the hospital and health bureau and the ministry of health. There is a need of creating awareness among nurses and midwives.

The response of participant: FGD4-07

I do not know the working time of the library. Some nurses and midwives do not know whether there is a library in the hospital or not. Moreover, when one leaves this hospital, he cannot get the library workers to the signature of clearance.

The response of participant: FGD4-08

I use training manuals and guidelines. I have not time to use books in the hospital. I use soft copy to read at home.

**5. How the supports for the implementation of evidence-based practice important?**

The response of participant: FGD4-05

There are quality unit officers, managers and non-governmental organizations. Quality unit health officers support the training of nursing care plans. Non-governmental organizations support our hospital on the training in family planning. However, there no stakeholders that support research utilization in clinical and healthcare practice

The response of participant: FGD4-08

There is not supportive supervision and controls of implementation of evidence-based practice of nurses and midwives

FGD5

1. **How do you perceive the implementation of Evidence-based Practice in your hospital? How do you understand the implementation of evidence-based practice?**

The response of participant: **FGD5-01**

My implementation of evidence-based practice is not satisfactory. I have experience of reading books for my implementation of evidence-based practice.

The response of participant: **FGD5-02**

I understand that evidence-based practice is the use of scientifically proved evidence in the health service. It is a means of clinical practice based on rules and follows the scientific procedure.

I know national guidelines used for the implementation of evidence-based practice. Research results are base for the implementation of evidence-based practice.

The response of participant: **FGD5-04**

Scientific sources of evidence, guidelines and hospital protocols are used for the implementation of evidence-based practice.

The response of participant: **FGD5-09**

Implementation of evidence-based practice is a professional obligation. I get the best sources of evidence from experienced health professionals. I got it from the health bureau and the ministry of health. We can also get research articles when we are interested but we do not use it for clinical decisions. I use guidelines prepared by seniors.

The response of participant: **FGD5-10**

I know the implementation of evidence-based practice. I have the perception. For example, when the doctor treats the patient based on guidelines, this is the implementation of evidence-based practice.

1. **How do you think about the importance of the implementation of evidence-based practice? What are pieces of evidence used for the implementation of evidence-based practice?**

The response of participant: **FGD5-01**

I implement evidence-based practice through reading documents and guidelines. I also ask seniors when I am not clear about some procedures. I understand the procedure from my colleagues. I use my colleagues as a source of knowledge and skills.

The response of participant: **FGD5-03**

Our ward uses treatment guidelines for the implementation of evidence-based practice. The other evidence is drug formulary. We get these guidelines from the health bureau. The guidelines are note updated every 5 years. The third evidence is senior health professionals. I saw general practitioners when they share new sources of evidence of research but they do not implement evidence-based practice unless seniors approve it.

The response of participant: **FGD5-04**

I am not quite sure about my implementation of evidence-based practice. However, the use of evidence or implementation of evidence-based practice improves ethical-related issues. Implementation of evidence-based practice is useful for the patients, nurses and midwives. Sharing of evidence among colleagues is important.

The response of participant: **FGD5-06**

I think the implementation of evidence-based practice improves the success of the clinical outcomes. I know other evidence used for the implementation of evidence-based practice.

1. **How do you think your / nurses’ and midwives’/ knowledge, skills, and attitude towards the implementation of evidence-based practice?**

The response of participant: **FGD5-07**

I have perception and knowledge about guidelines’ use for evidence-based practice. We do not read research articles for the sake of clinical decisions. Our hospital staff members use guidelines for the implementation of evidence-based practice. The senior doctors tell us about research results to implement evidence-based practice. However, I do not have the knowledge and the skill to use research findings as a source of knowledge and skill

1. **Why don’t you implement evidence-based practice?**

The response of participant: **FGD5-02**

There are barriers to the implementation of evidence-based practice. These are lack of access to guidelines, the internet, and other evidence. I do not use the internet because it harms my eye. The other barrier is the hospital library is far from the ward. Moreover, it has no space and tables for reading materials in the library. Many people cannot use the library at the same time.

Experienced colleagues are limited to their ward. There is a rotation of staff every year. when experienced staff works in one ward for a long period, he/she become master for that particular activity. Because of rotation, the staff members lose their experience and they are like fresh for their new ward. The other problem is lack of attitude and motivation of staff to share their experience.

1. **How the supports for the implementation of evidence-based practice important?**

The response of participant: **FGD5-01**

I know that there is managers’ support regarding the implementation of evidence-based practice. However, there are different challenges of manager’s support. For example, salary improvement and incentives are beyond manager’s activities.

The response of participant: **FGD5-05**

There is no managerial support for the implementation of evidence-based practice in our hospital. The manager and heads follow which activity is well done and which one is not well done. They do not know what is important for the implementation of evidence-based practice during clinical decisions. There is no way to address the implementation of evidence-based practice.

The response of participant: **FGD5-06**

Sources of evidence used for the clinical decisions should be available. For example, drug information center should be established. Internet access should be available in all wards. Nurses’ and midwives’ commitment is important despite the accessible sources of evidence. Managers and heads should introduce the use of implementation of evidence-based practice through training and discussion during a staff meeting. Managers and heads supervise and control nurses and midwives’ implementation of evidence-based practice.

I know that managers and heads do not supervise and control our implementation of evidence-based practice.

**Interview**

1. **How do you think about the implementation of evidence-based practice in your hospital? How do you perceive IEBP?**

The response of the interviewee: **101**

I think, there is a low implementation of evidence-based practice in our hospital. This does not mean that nurses and midwives do not implement evidence-based practice.

When I see my work experience in this hospital, there is the handling of sources of evidence like guidelines.

1. **How do you think about the importance of the implementation of evidence-based practice? What are important sources of evidence used for the implementation of evidence-based practice?**

The response of the interviewee: **101**

Nurses and midwives use guidelines and standards of the ministry of health. However, we do not use updated books and research articles.

1. **What are the reasons that you do not use evidence like research, standard guidelines, hospital protocols, books for your healthcare and clinical decision-making practice?**

The response of the interviewee: **101**

Our hospital library is small. It has no space to read books and other sources of evidence used for the implementation of evidence-based practice. It is not comfortable. In addition to this, there is a shortage of time due to the workload of nurses and midwives.

There are no job descriptions of MSc, BSc, and diploma nurses and midwives. There is no clear demarcation of job descriptions among nurses and midwives based on the level of education. You see here, this is a matter of moral. I feel less interested when I am always doing the same job as a diploma.

1. **How the supports for the implementation of evidence-based practice important?**

The response of the interviewee: 101

The manager and ward heads do not supervise and control nurses’ and midwives’ implementation of evidence-based practice using guidelines, research articles and books.

I tried to solve the problem by preparing shelves to place guidelines and other sources of evidence. I place training manuals and guidelines on the shelf. I request the manager to get a clear job description and write letters to train newly engaged midwives. However, the response is not satisfactory. Relating use of books and research articles, midwives use sources of evidence that they have on hand but nothing is done to use research articles in our hospital.

I ask responsible bodies for the implementation of evidence-based practice like a pediatric ward, OR head and chief managers. I did nothing beyond this. I cannot communicate outside the hospital. In our hospital, without higher officials departments cannot communicate with other stakeholders. The chief manager can do this.

There is experience and evidence sharing among colleagues. There are guidelines in my ward. However, we do not use sources of evidence to implement evidence-based practice.

1. **How do you think about the implementation of evidence-based practice in your hospital? How do you perceive IEBP?**

Implementation of evidence-based practice is a means of applying knowledge and skills in clinical and healthcare practice using the best source of knowledge and skills.

The response of the interviewee: **102**

1. **How do you think about the importance of the implementation of evidence-based practice? What are important sources of evidence used for the implementation of evidence-based practice?**

The response of the interviewee: **102**

In any health institution, providing health care service based on the implementation of evidence-based practice is important. I think the community can get quality health services by the implementation of evidence-based practice. Equivalent health service for the increased need of the community for health care is impossible without the implementation of evidence-based practice. Health care service can not satisfy the community without the implementation of evidence-based practice. The success of health care is the result of the implementation of evidence-based practice.

By now internet access is available in all wards. There is an internet connection in all departments. Nurses and midwives google when they want to read. There is a way to get national and international references like research articles. However, there is less use of research findings for the implementation of evidence-based practice.

1. **What are the reasons that you do not use evidence like research, standard guidelines, hospital protocols, books for your healthcare and clinical decision-making practice?**

The response of the interviewee: **102**

This is the impact of lack of internet access and library. Nurses and midwives use standard guidelines and hospital protocols for the implementation of evidence-based practice

I believe that the guidelines of the ministry of health are not enough. There are newly updated research results. However, newly updated researches are not used in our hospital.

Most of the time, our focus is on the latest activities requested by the health bureau and the ministry of health and activities that call us for competition among institutions. However, there is no system for the implementation of evidence-based practice. The second factor is the absence of nurses’ and midwives’ job descriptions. For example, there is no clear demarcation of jobs among MSc, BSc, and diploma nurses and midwives.

The third factor is lack of incentive. Due to lack of incentives, nurses and midwives lack motivation. They are not interested to devote their time through reading to implement evidence-based practice. The fourth factor is managers and we heads do not fulfill internet access, functional library and other logistics. The last factor is nurses’ and midwives’ workload.

There are factors like lack of interest. Internet access is not still accessible at all levels.

1. **How the supports for the implementation of evidence-based practice important?**

The response of the interviewee: 102

We did not conduct the assessment of the implementation of evidence-based practice of nurses and midwives exactly. We did not know their implementation of evidence-based practice. However, there are competency tests to conduct supportive supervision.

We have an onsite training program for newly engaged staff members. There are newly launched standards of nursing and midwifery care. We conducted the assessment based on this standard and identify gaps. We provide intervention for the identified gaps in the form of training. There is also the morning session including doctors on Monday and Friday. During the morning sessions, the staff shares knowledge that is used for the implementation of evidence-based practice.

1. **How do you think about the implementation of evidence-based practice in your hospital? How do you perceive IEBP?**

The response of the interviewee: **103**

We know nurses’ and midwives’ implementation of evidence-based practice through evaluation and observation. When we see vaccine service, first nurse or midwife get training, then we evaluate performance. If there is a gap, we consider that there is no implementation of evidence-based practice.

1. **How do you think about the importance of the implementation of evidence-based practice? What are important sources of evidence used for the implementation of evidence-based practice?**

The response of the interviewee: **103**

Most of the time nurses and midwives use national guidelines for the implementation of evidence-based practice. However, they do not use it always. We cannot get research articles in our hospital and we cannot use it. We can get research from the internet but there is no internet access in our hospital. We use senior nurses and midwives for the implementation of evidence-based practice through sharing their experiences.

1. **What are the reasons that you do not use evidence like research, standard guidelines, hospital protocols, books for your healthcare and clinical decision-making practice?**

The response of the interviewee: **103**

Some factors hinder our implementation of evidence-based practice for nurses and midwives. There is a shortage of books and guidelines. The other is the lack of nurses’ and midwives’ perceptions.

1. **How the supports for the implementation of evidence-based practice important?**

The response of the interviewee: 103

There is not supportive supervision and control of nurses and midwives’ implementation of evidence-based practice. We will do this in the future. Implementation of evidence-based practice is good. It protects us from accountability.

I know stakeholders like hospital managers, heads of the woreda health office, non-governmental organizations (NGOs) and others that can support the implementation of evidence-based practice. NGOs provide us the guidelines and training manuals. Other stakeholders do nothing to implement evidence-based practice. I did not communicate with any stake holders.

As the head of the ward, I am finding getting guidelines, books and research articles and internet access. I have not done this. After nurses and midwives get training, everyone shares knowledge, skill and manuals obtained from training. We do nothing other than this to implement evidence-based practice.

1. **How do you think about the implementation of evidence-based practice in your hospital? How do you perceive IEBP?**

The response of the interviewee: **104**

Including doctors, all nurses and midwives use books, guidelines and hospital protocols for the implementation of evidence-based practice. However, I do not think all nurses and midwives use these sources of evidence for the implementation of evidence-based practice. It is impossible to say most nurses and midwives do not implement evidence-based practice. Nurses and midwives use guidelines and standards for the implementation of evidence-based practice. We evaluate the implementation of evidence-based practice quarterly. We use the nursing audits to evaluate the implementation of evidence-based practice. We provide feedback for nurses and midwives based on the audit.

1. **How do you think about the importance of the implementation of evidence-based practice? What are important sources of evidence used for the implementation of evidence-based practice?**

The response of the interviewee: **104**

Implementation of evidence-based practice is important for quality healthcare services and patient satisfaction. If patients are not satisfied, they find getting good service to other health institutions. Therefore, the implementation of evidence-based practice is mandatory.

Implementation of evidence-practice is evaluation criteria for nurses and midwives in our hospital. There is the ministry of health direction that allows the use of all trusted sources of evidence for nurses’ and midwives’ implementation of evidence-based practice. Important sources of evidence can be reported to hospital management and used as a benchmark even for clustered hospitals.

There are guidelines and other sources of evidence used for the implementation of evidence-based practice. There are 20 chapters for nurses and midwives in the reform. According to the reform, nurses and midwives can prepare their guidelines and they are autonomous to have their policies. Nurses and midwives have 10 standards and operational standards. These standards are prepared and set in all departments.

There should be access to use research articles and other sources of knowledge and skills. If there is access, we can use different guidelines other than national guidelines. There is a challenge or limitation to use sources of evidence because of assistance nurses cannot understand through reading. There is a nursing care plan and nursing policy. There is the training of nurses and midwives in the cluster to use guidelines, policies and nursing care plans. There are nursing intensive care unit standard guidelines, emergency guidelines, midwives standard guidelines and other standard guidelines. Leader of clustered hospitals provide training about these standards and supervise hospitals quarterly to identify gaps for supportive intervention.

1. **What are the reasons that you do not use evidence like research, standard guidelines, hospital protocols, books for your healthcare and clinical decision-making practice?**

The response of the interviewee: **104**

National guidelines are distributed for new hospitals recently. These guidelines should be distributed before the launch of health care services. However, we can get guidelines from our mobile internet. For example, I use my mobile to download around 50 books to use them for clinical decisions. We download these sources of evidence and put the evidence on our desktop.

Nurses and midwives do not implement evidence-based practice because of lack of access to materials, nurses and midwives stick to traditional practice, negligence, lack of incentive, and lack of managerial support. Lack of material access is one of the challenges in our hospital. The hospital is established recently. We buy materials for fetching water and internet card to download books, guidelines and others by taking money from our pocket. There is no bed and laundry. If more patients come for operation, we do nothing. These all challenges have an impact on the implementation of evidence-based practice

1. **How the supports for the implementation of evidence-based practice important?**

The response of the interviewee: 104

There is an established group for team management. The nursing team management prepares guidelines. There is also a selected supervision committee. I am the head of nursing supervisors and we supervise nurses and midwives according to the schedule using a checklist. Based on the identified gaps during supervision, here is a supportive intervention.

Non-governmental organizations are the only stakeholder to support the implementation of evidence-based practice. I did nothing to communicate with stakeholder about the implementation of evidence-based practice.

1. **How do you think about the implementation of evidence-based practice in your hospital? How do you perceive IEBP?**

The response of the interviewee: **105**

I think nurses and midwives have the perception of the implementation of evidence-based practice because of their many endeavors to improve their performance. Most of them are young. They are interested to improve their knowledge. They have a good attitude but we haven’t measured knowledge during observation.

1. **How do you think about the importance of the implementation of evidence-based practice? What are important sources of evidence used for the implementation of evidence-based practice?**

The response of the interviewee: **105**

Implementation of evidence-based practice is very useful. As you know, medicine is updated every time. Evidence that we use today may not work for tomorrow.

Nurses and midwives should use guidelines and hospital protocols without missing. This is very important for the knowledge and skill of nurses and midwives. Short-term and long-term training are also important. The training should be through identifying gaps for the intervention. There should be knowledge transformation.

Some activities of medicine do not have guidelines. Few procedures have national guidelines. It is impossible to address all medical activities using guidelines. When I see practically, there are communicable disease control, maternal and child treatment guidelines. There are not non-communicable disease guidelines. Nurses and midwives use their knowledge and other evidence for the implementation of evidence-based practice. Nurses and midwives can use any evidence for the implementation of evidence-based practice. There is no prohibition to use evidence. However, our implementation of evidence-based practice should be safe for the patient.

Nurses and midwives are dependent on the national guidelines. This is because of clarity and shortage of guidelines. Sometimes, there may be different guidelines and difficult to use the best one. In this situation, nurses and midwives should ask their seniors to use the best evidence for the implementation of evidence-based practice. The other problem is getting the evidence lately especially in primary hospitals. Due to this, nurses and midwives use outdated evidence.

I know sources of evidence used for nurses and midwives very well. If you ask me to mention, there are guidelines, hospital protocols and others. We usually distribute these sources of evidence and I understand very well.

1. **What are the reasons that you do not use evidence like research, standard guidelines, hospital protocols, books for your healthcare and clinical decision-making practice?**

The response of the interviewee: **105**

Nurses and midwives do not implement evidence-based practice because of lack of commitment, lack of motivation, shortage of availability of evidence, lack of managers and heads support, uncomfortable workplace, lack of availability of materials, lack of internet access, and lack of library personnel in the hospital.

There is not human resource model 15 for district hospitals. There is no library personnel. There are books in our store. The hospital has no library. There are no constructed rooms and library personnel. Some nurses use the internet for social media and self-entertainment instead of using it as sources of evidence for professional knowledge.

1. **How the supports for the implementation of evidence-based practice important?**

The response of the interviewee: 105

There are NGOs like CDC, World Vision and Gender Health Ethiopia that provide short-term training and different guidelines. These organizations did well but it is not adequate to support IEBP. We use these updated guidelines. Nevertheless, there is a big gap in using research articles and there are no supporters to use it.

Our hospital has different checklists. There is a quarterly evaluation based on Ethiopian transformation guidelines. One evaluation criteria is the availability of guidelines and hospital protocols to wards and departments. The second criterion is quality standard. There is a quality standard means nurses and midwives use guidelines, hospital protocols and other evidence. In addition to this, we have three morning sessions per week that help us to shares knowledge during morning session discussions.

The hospital has an evaluation system. The quality standard of the hospital is evaluated by Ethiopian health sector transformation guidelines. However, there are not evaluation standards for the implementation of evidence-based practice. There is not supportive supervision of the implementation of evidence-based practice of nurses and midwives.

As case manager, I assign hospital worker who has easy work so that at least they can borrow books and guidelines for nurses and midwives. The second one is I tried to avail broadband internet access in the hospital and there is internet service

When new standards and activities are launched from the ministry of health, the manager and ward heads introduce the implementation of evidence-based practice for nurses and midwives. There is a way to share knowledge and skill for nurses and midwives. In addition to this, soft copies are available in each ward. There is also one to fife discussion session for nurses and midwives that help them to share knowledge and skill.

1. **How do you think about the implementation of evidence-based practice in your hospital? How do you perceive IEBP?**

The response of the interviewee: **106**

I know the implementation of evidence-based practice. It is the use of different sources of evidence to update knowledge and skills. It is a matter of reading textbooks, standard guidelines, national protocols and research findings.

1. **How do you think about the importance of the implementation of evidence-based practice? What are important sources of evidence used for the implementation of evidence-based practice?**

The response of the interviewee: **106**

Nurses and midwives implement evidence-based practice using national guidelines, hospital protocol, research articles and other sources of evidence based on the principles when they provide health services. There is sharing of knowledge and skills among nurses, midwives and doctors. There is the implementation of evidence-based practice in our hospital. I think there could be a gap in nurses’ and midwives’ implementation of evidence-based practice. There are no adequate guidelines, research articles, and internet access.

1. **What are the reasons that you do not use evidence like research, standard guidelines, hospital protocols, books for your healthcare and clinical decision-making practice?**

The response of the interviewee: **106**

Nurses and midwives do not implement evidence-based practice because of lack of knowledge, negligence, lack of attitude and lack of understanding about sources of evidence standard guidelines updated books and research findings. The hospital has no books.

1. **How the supports for the implementation of evidence-based practice important?**

The response of the interviewee: 106

We checked the implementation of evidence-based practice during weekly, monthly and quarterly meetings. First, Nurses and midwives announce their sharing of evidence-based practice among themselves. Second, we assess nurses’ and midwives’ use of guidelines in the ward. We identify nurses’ and midwives’ implementation of evidence-based practice using guidelines in one to five discussions.

The hospital has a committee to evaluate the implementation of evidence-based practice using guidelines and hospital protocol. The committee members evaluate the availability of guidelines and hospital protocols quarterly. The evaluation identified that implementation of evidence-based practice is low in some wards and good in the other wards. We have no access to research articles due to a lack of internet access.

Nurses and midwives got on-site training to implement evidence-based practice. I supported them about sharing evidence among nurses and midwives. They got a benchmark for experience sharing.

I facilitate training for nurses and midwives in collaboration with Debre Tabor University, the health bureau and other stakeholders. The trained nurses and midwives share the knowledge, skills and manuals obtained from the training.

1. **How do you think about the implementation of evidence-based practice in your hospital? How do you perceive IEBP?**

The response of the interviewee: **107**

I should understand the implementation of evidence-based practice. I do not think that I have a full understanding of how to implement evidence-based practice. I have the idea but the confidence to do exactly.

1. **How do you think about the importance of the implementation of evidence-based practice? What are important sources of evidence used for the implementation of evidence-based practice?**

The response of the interviewee: **107**

We can judge the trustfulness of the sources of evidence other than guidelines and hospital protocols by using our previous knowledge and scientific explanation. We can also share new evidence or sources of knowledge and skills from seniors.

Our hospital is different from other hospitals for its implementation of evidence-based practice. For example, we knew the reduction of partner tests through conducting research. There was a project proposal to solve the identified problems. There are few nurses and midwives who use guidelines and research articles for the implementation of evidence-based practice. Most nurses and midwives use knowledge and skills from training for the implementation of evidence-based practice.

There is a culture of sharing of knowledge and skills among nurses and midwives to implement evidence-based practice.

1. **What are the reasons that you do not use evidence like research, standard guidelines, hospital protocols, books for your healthcare and clinical decision-making practice?**

The response of the interviewee: **107**

Nurses and midwives know sources of evidence like the internet. However, some factors hinder the implementation of evidence-based practice. The first one is work overload. Health care is becoming forceful activity. Nurses and midwives become tired due to work overload. When they are tired, they do complete the patient card even.

Guidelines are placed in each working room. However, nurses and midwives do not use it because of a lack of commitment and lack of experience to read guidelines for the implementation of evidence-based practice. Work overload is the most influencing factor in the implementation of evidence-based practice in our hospital. There is a lack of internet access to get sources of evidence like research articles.

I also think that there are few nurses and midwives who have the problem of knowledge, skills and attitude. In addition to this, there is a lack of confidence of nurses and midwives during their practical clinical decision. This is because of the fear of senior doctors. There is also an inferiority complex between nurses and doctors and midwives and doctors to implement evidence-based practice. Nurses and midwives are not competent when they are graduated from colleges and universities. Some of them do not perform even simple procedures.

Nurses and midwives are not interested in the on-site training. Practically, they are not happy to get training in the hospital. There is also a lack of attitude. I do not think that nurses and midwives use research result to implement evidence-based practice.

1. **How the supports for the implementation of evidence-based practice important?**

The response of the interviewee: 107

There is no system of supportive supervision and controls of implementation of evidence-based practice among nurses and midwives in our hospital. But I tried to share confidence on how to consult cases for seniors. I shared my knowledge and skill of family planning with my colleagues after I had got training.

I got stakeholders like hospital manager and non-governmental organization like nursing association

We did well about the use of hospital protocol to implement evidence-based practice. There is one to five government structure in this hospital. During this session, there is sharing of ideas and knowledge about the use of hospital protocols. As a department, there is sharing of knowledge, skills and how to use the evidence.

1. **How do you think about the implementation of evidence-based practice in your hospital? How do you perceive IEBP?**

The response of the interviewee: **108**

Most of the health care management is based on seniors. Some of the experienced senior nurses and midwives are good and others are not good. Nurses and midwives are far from sources of knowledge and skills, unless they are academic staff use sources of evidence. Therefore, sources of evidence should be updated for the implementation of evidence-based practice.

1. **How do you think about the importance of the implementation of evidence-based practice? What are important sources of evidence used for the implementation of evidence-based practice?**

The response of the interviewee: **108**

We have guidelines and manuals. However, implementation of evidence-based practice is low. Nurses and midwives always use training manuals for the implementation of evidence-based practice. They do not use research articles for the implementation of evidence-based practice. For example, I got research articles that were done in Egypt and Malawi. The finding of the studies indicated that cleaning of perineum using an antiseptic before the vaginal examination prevent infection. I post the abstract on the board. Previously, nurses and midwives used antiseptic solutions for cleaning of perineum before a vaginal examination. They do not use antiseptic solution during vaginal examination at present. They haven’t implemented evidence-based practice.

When newly graduated nurses and midwives share experiences from seniors who use only their experience. This is also a challenging issue. I do not think that all nurses and midwives use sources of evidence for the implementation of evidence-based practice. Those who got training and training manuals implement evidence-based practice. However, I do not read research articles and updated standard guidelines.

We use experience in healthcare and clinical decision. Once, graduate from college, we continue to use our experience without using scientific evidence

1. **What are the reasons that you do not use evidence like research, standard guidelines, hospital protocols, books for your healthcare and clinical decision-making practice?**

The response of the interviewee: **108**

We do not implement evidence-based practice because of different factors. Lack of reading the latest sources of evidence after graduation prevents the implementation of evidence-based practice. We use our knowledge and skills obtained before graduation. We use old knowledge for healthcare and clinical decisions. There is no responsibility for nurses’ and midwives’ use of recent evidence in the system of our hospital.

Nurses and midwives do not read sources of evidence like books, articles, guidelines and hospital protocols. They care about reading because of a lack of incentive. There is attitude problem. The knowledge of nurses and midwives is not similar. Nowadays, graduate nurses and midwives different from colleges and university have different knowledge and skill. Some of these nurses and midwives do not know even simple procedures. However, they improve their practice by observing their seniors.

There is no workload in our hospital. The hospital is a primary hospital. It is different from referral hospitals which have nurses’ and midwives’ workloads.

There was a library in our hospital but there is a shortage of room and it was shifted to a drug information center. There are books in the drug information center room. Around one to two percent of nurses and midwives read these books.

There is internet access in our hospital but most nurses and midwives use it for social media. There is no habit of reading medical articles. Unless ministry of health and the health bureau should have a system of reading sources of evidence for nurses and midwives. In the present situation, the implementation of evidence-based practice is very difficult.

We have not done sharing knowledge and skill formally among nurses and midwives. To tell the truth, the staff did not present during the morning meeting. We take attendance. By now, I take the measure of withholding 3 days absentees from duty and all the staffs attending morning session without missing.

Generally, implementation of evidence-based practice is a very great job. Other countries implement evidence-based practice. The senior professionals are interested in sharing the experience with juniors without updating themselves. Juniors are interested in evidence-based practice. This kind of situation leads to a superiority complex**.** Some old staffs use knowledge and skill that they get from school to do their business. They do not have reading habits.

Let me tell you the real history of an educated patient and doctor. The doctor has been a long time in the hospital. He was frustrated and he was as he had been graduated from college. There was no incentive for him to do his job and negligence is his habit which does not lead to a good attitude. The patient knows the doctor very well. The patient got this doctor during the examination, and the patient said that no…he did not read anything about his profession after he had been graduated. The patient went to another doctor who was working in the university thinking that he read many books and articles. Similarly, nurses and midwives were not motivated to update themselves through reading books, guidelines, and research articles. They stick to traditional practice.

**How the supports for the implementation of evidence-based practice important?**

The response of the interviewee: 108

The ministry of health should incorporate the latest research articles and other sources of evidence in the national guidelines. The updated sources of knowledge and skills should be introduced for nurses and midwives. There is no supportive supervision in our hospital and this should be considered.

There are no stakeholders for the implementation of evidence-based practice. I have prepared notes, PowerPoint and download articles and print these sources of evidence to place in each ward so that nurses use these sources of evidence for the implementation of evidence-based practice.

I prepared seminar presentations and PowerPoint for nurses and midwives. This is not continued. I did a weekly seminar program and I put evidence in the ward. Some of them read the sources of evidence and others did not use these materials. We also placed hospital protocols in the ward.

There are representatives of quality health care in the hospital and health bureau. The quality unit representative is not independent. It is the extra activity of the representative. The quality health care office should include the implementation of evidence-based practice.

1. **How do you think about the implementation of evidence-based practice in your hospital? How do you perceive IEBP?**

The response of the interviewee: **109**

I cannot determine how nurses and midwives implement evidence-based practice. We do not have time table when to use sources of evidence. However, I understand that we implement evidence-based practice all the time when patients visit the hospital to get our services. We use guidelines to help us in clinical and healthcare practice

1. **How do you think about the importance of the implementation of evidence-based practice? What are important sources of evidence used for the implementation of evidence-based practice?**

The response of the interviewee: **109**

The use of guidelines determines better health services especially for antenatal care ( ANC), family planning and other services. Implementation of evidence-based practice is mandatory for patient satisfaction and it helps nurses and midwives to provide quality health care without difficulty. I think nurses and midwives use guidelines for the implementation of evidence-based practice

We haven’t used research articles to implement evidence-based practice. The use of the research article is incomplete. There is not any guideline in the outpatient department (OPD). However, there are research articles that we use in the tuberculosis (TB) room. We use these articles to provide service without difficulty. Sometimes, we do not implement evidence-based practice using sources of evidence like guidelines unless we get training, guidelines and manuals

All professionals have the knowledge that they got from their college and university. For example, nurses and midwives provide malaria management services using their knowledge without any sources of evidence like guidelines. If they know, they can provide the service without evidence. I tried to get guidelines and manuals when I went to zones and the health bureau for a meeting. In addition to this, when nurses and midwives got training, we had a meeting to share knowledge of the training for all staff members.

Evidence used for the implementation of evidence-based practice is different from ward to ward or department to department. For example, if you take outpatient department nurses, they use evidence from the internet.

We use guidelines and manuals by searching on the internet. However, there is internet access interruption. Moreover, we had a morning session and a case presentation to learn from each other. During the meeting, the discussion is about health services provided for the patient, guidelines and other sources of evidence. All members of the meeting can state whether they implement evidence-based practice or not.

1. **What are the reasons that you do not use evidence like research, standard guidelines, hospital protocols, books for your healthcare and clinical decision-making practice?**

The response of the interviewee: **109**

Internet access was started in our hospital. Therefore, we search updated textbooks, standard guidelines, research articles and national guidelines for the implementation of evidence-based practice easily. By the way, our internet access is limited in our hospital. However, it is useful when data internet access is closed.

We do not implement evidence-based practice because of a lack of guidelines, lack of training and weak communication among nurses and midwives. There is also a factor like lack of participation during the morning sessions.

If guidelines are available on the website of the ministry of health, we can place the guidelines and other sources of evidence in each ward and department. The other barrier is nurses’ and midwives’ negligence. This negligence is because of attitude. There could be a lack of knowledge and skill. It depends on individual differences. I do not think there could be a significant difference because the gap of nurses and midwives intervened through training and morning sessions. Management support is also a factor for the implantation of evidence-based practice. Nurses’ and midwives’ dissatisfaction because of lack of incentive leads to negligence. The training program was withheld by the manager when nurses and midwives got training opportunities in our hospital. This is a cause of negligence.

There is a small class for reading books. The kooks are few. We do not use these sources of evidence for the implementation of evidence-based practice.

1. **How the supports for the implementation of evidence-based practice important?**

The response of the interviewee: 109

We have supportive follow up during morning sessions and other meetings. During mentoring, nurses and midwives fill their gaps. However, there is no planned supportive supervision and controls of implementation of evidence-based practice in this hospital

As I told you before, there is a disease difference from place to place. Ten top diseases in Gayint are different from other places. Therefore, the ministry of health should avail the sources of evidence like guidelines based on the diseases present in a specific place.

I did bring guidelines and manuals from the zone and health bureau when I went to a meeting.

There is work overload in the outpatient department. Nurses and other professionals do not write patient assessments and reports. I also did this. Therefore, it is better to decrease patients per service provider to implement evidence-based practice. We also have to create awareness of nurses and midwives to implement evidence-based practice

1. **How do you think about the implementation of evidence-based practice in your hospital? How do you perceive IEBP?**

The response of the interviewee: **110**

I do not think that there is a problem with knowledge, skill and attitude. Nurses and midwives are updating themselves by improving their level of education. There are training manuals and guidelines. For example; we have abortion guidelines and prevention of mother-to-child transmission (PMTCT). When nurses and midwives get manuals and training, they share the knowledge and skills for those who do not get the training. We place guidelines and manuals in the ward. There is sharing of sources of evidence among nurses and midwives during the meeting. We also have seminar presentations and morning session discussions in our hospital including our ward. I think this is the implementation of evidence-based practice.

1. **How do you think about the importance of the implementation of evidence-based practice? What are important sources of evidence used for the implementation of evidence-based practice?**

The response of the interviewee: **110**

I work in the gynecology ward. We have guidelines. Most of the time, we use abortion guidelines. Many nurses and midwives came from other hospitals and they have work experience. They share the experience. Most gynecology procedures do not have guidelines. We use seniors as a source of knowledge and skills for the implementation of evidence-based practice.

When the new nurses and midwives join this hospital, the previous senior nurses and midwives share their experiences with the newcomers. We also have books and reading rooms. Nurses and midwives update themselves through reading.

1. **What are the reasons that you do not use evidence like research, standard guidelines, hospital protocols, books for your healthcare and clinical decision-making practice?**

The response of the interviewee: **110**

There is a high patient flow to this ward. We cannot read books in our ward. Patient flow and the number of nurses and midwives are not balanced. The staff members are few and patient flow is very high.

Nurses and midwives do not implement evidence-based practice because of a shortage of time due to work overload, lack of access to sources of evidence, lack of updated evidence, lack of library, long distance of home from the hospital, lack of internet access in the ward. You see this ward. You cannot go far distance to get internet from this ward. There is no service of the bus for nurses and midwives. Our home is far from the hospital. It wastes our time to read guidelines, books, research articles and so on.

1. **How the supports for the implementation of evidence-based practice important?**

The response of the interviewee: 110

There is no managers and other stakeholder support for the implementation of evidence-based practice.

1. **How do you think about the implementation of evidence-based practice in your hospital? How do you perceive IEBP?**

The response of the interviewee: **111**

I think most nurses and midwives have well experiences to implement evidence-based practice. I do not consider that all experienced nurses and midwives implement evidence-based practice. We usually use nurses’ and midwives’ experiences for the implementation of evidence-based practice. We share knowledge and skills from our experience for the implementation of evidence-based practice.

1. **How do you think about the importance of the implementation of evidence-based practice? What are important sources of evidence used for the implementation of evidence-based practice?**

The response of the interviewee: **111**

We have many experiences in our hospital. Doctors also have many experiences. We have one to five group discussions to share our experience.

We use the internet to get evidence for the implementation of evidence-based practice. I think our nurses are interested to share their experiences and get knowledge from others.

1. **What are the reasons that you do not use evidence like research, standard guidelines, hospital protocols, books for your healthcare and clinical decision-making practice?**

The response of the interviewee: **111**

We do not have any plan of implementation of evidence-based practice because of emergency activities. We work for 24 hours. We are too busy. We can get different pieces of evidence from the library. However, we cannot go to the library due to a lack of time in this emergency ward. Nurses are few compared to emergency activities. Nurses are working continuously. They are strong. Nurses do not use evidence because of the workload. As I told you, we have a shortage of time. I have no time to read books. There is tiredness. We sleep, when we get time. We cannot consider anything rather than this because of a shortage of time.

We do not have a broad program for the implementation of evidence-based practice because of emergency activities. We work for 24 hours. We are too busy. Nurses and midwives of other wards use updated sources of evidence for the implementation of evidence-based practice. They give us books to use for the implementation of evidence-based practice.

We do not use books for the implementation of evidence-based practice. There are not updated guidelines in our ward. There are not research articles and hospital protocols for this ward.

We can get different sources of evidence from the library. However, we cannot go to the library due to a lack of time in the emergency ward. There are few nurses compared to emergency activities.

We do not implement evidence-based practice because of workload, lack of material access like internet, guidelines and hospital protocols. I think there is not lack of knowledge, skill and attitude in our hospital.

We do not have books in our emergency ward. We have no library and we have to borrow from colleagues to get books. As I told you, we have a shortage of time. I have not any time to read books. There is tiredness. we tried to sleep, when we get time. We cannot consider anything rather than this because of a shortage of time.

1. **How the supports for the implementation of evidence-based practice important?**

The response of the interviewee: 111

There is not supportive supervision and control of implementation of evidence-based practice using research articles, guidelines, hospital protocols and books.

1. **How do you think about the implementation of evidence-based practice in your hospital? How do you perceive IEBP?**

The response of the interviewee: **112**

Most of the midwives in this hospital are newly employed. We were recently graduated from colleges and universities. We know to implement evidence-based practice. In addition to this, we share the experience with experienced nurses and midwives especially the experience of activities of operation room scrub nursing and waiting nurse services.

1. **How do you think about the importance of the implementation of evidence-based practice? What are important sources of evidence used for the implementation of evidence-based practice?**

The response of the interviewee: **112**

Regarding guidelines, there is a magnesium sulphate protocol that is posted in the delivery ward. We use this when we administer magnesium sulphate. There is also a protocol for retroviral infection (RVI) patients. Newly engaged nurses and midwives share the experience of neonatal resuscitation procedures from residences and senior midwives. Previously, there was a computer desktop in our ward. We had soft copies of books, guidelines and other evidence on the desktop. However, there are not any books in our ward.

1. **What are the reasons that you do not use evidence like research, standard guidelines, hospital protocols, books for your healthcare and clinical decision-making practice?**

The response of the interviewee: **112**

I do not have a clear order of implementation of evidence-based practice of nurses and midwives. I do nothing about the use of research articles for the implementation of evidence-based practice rather than being a study participant.

There is internet access even in our ward. However, nurses and midwives forget their practice because of wrong use of the internet like chatting face book and following social media instead of using the internet for searching best evidence in clinical practice. Nurses and midwives should use the internet for the benefit of patients.

We had morning sessions and discussion sessions based on selected cases. This was not continued. There is the rotation of midwives and nurses from ward to ward. The morning session was failed because of rotation. The newly rotated midwives need adaption to the ward.

Nurses and midwives do not implement evidence-based practice because of work overload.

Nurses and midwives work all day without rest and they become tired. When we go home, we want to sleep because of tiredness. We are not motivated to read the different sources of evidence like books, guidelines, research articles and other evidence. When we get uncommon cases, we use our mobile to get sources of evidence from the internet. We do not use the library to read and refer books. We have no plan to implement evidence-based practice.

There is the problem of competency when nurses and midwives were graduated from their colleges and universities. For example, one professional inserts a catheter through the vagina instead of the urethra to catheterize the patient. I think when he was a student; he should practice the demonstration of catheterization. Unless he was competent, he should not have been graduated

I know there is no in-service training program for nurses and midwives by this time. We do not have national guidelines. Managers and matrons do not bother about guidelines. There is no awareness of the implementation of evidence-based practice.

1. **How the supports for the implementation of evidence-based practice important?**

The response of the interviewee: 112

What do you mean? How could we change without the mentoring and supervision of the IEBP? I understand that it is important. However, there is no direct mentoring and supportive supervision, control and evaluation of nurses’ and midwives’ use of guidelines, books, and manuals. We do it indirectly. I do not expect something good about the use of research in our clinical settings.

There is not direct supportive supervision and control of nurses’ and midwives’ implementation of evidence-based practice.

I have not got any stakeholders working in the implementation of evidence-based practice. We have not authority to communicate with non-governmental organizations. I did nothing about the implementation of evidence-based practice. I communicate to my senior heads about in-service training.

1. **How do you think about the implementation of evidence-based practice in your hospital? How do you perceive IEBP?**

The response of the interviewee: **113**

It is getting reading materials such as books, articles, updated and standard guidelines and national protocols.

1. **How do you think about the importance of the implementation of evidence-based practice? What are important sources of evidence used for the implementation of evidence-based practice?**

The response of the interviewee: **113**

Regarding national guidelines, there are launched activities. These sources of evidence are important to keep quality health care. There are many standards in our hospital currently. These are Ethiopian implementation guidelines, clean and safe hospital standards and other standards. Our hospital uses these guidelines and standards strongly.

1. **What are the reasons that you do not use evidence like research, standard guidelines, hospital protocols, books for your healthcare and clinical decision-making practice?**

The response of the interviewee: **113**

We do not implement evidence-based practice because of a lack of reading habits, and unsuitable health systems to use evidence. When I consider this, our setup is not good or comfortable. For example, there is a lack of library, shortage of internet access and lack of other material access. The other barrier is work overload.

Nurses provide nursing care. However, nursing activities become physical activities. We work when doctors order treatment only. Nurses and midwives do not apply their creativity and they do not update themselves to provide quality health care.

Nurses and midwives do not read evidence because of lack of time, a gap of knowledge, skills and attitude. If we update ourselves through reading, nurses and midwives provide quality health care. This is important for health service providers, patients and hospitals.

There is no problem with knowledge and attitude. However, there is a skill gap among nurses and midwives. There is a theory-based education system in our colleges and universities. When we see practical skills, there is a problem with being competent. Fresh nurses and midwives cannot perform their activities. We fill this gap using senior nurses and midwives as a peer reviewer to support newly graduated nurse and midwives

Health care without nurses and midwives is meaningless. There is no plan for nursing and midwifery activities. There is not budget planning for training of nursing activities

1. **How the supports for the implementation of evidence-based practice important?**

The response of the interviewee: 113

There is no planned supportive supervision and control of the implementation of evidence-based practice. However, there are some activities that supervise and control the implementation of evidence-based practice indirectly. For example, if there is new event, there is discussion during the morning sessions and there is sharing of the best activities.

There are many stakeholders in our hospitals. I am one of the stakeholders as a quality unit leader. There are team leaders in each ward. There are also non-governmental organizations. These organizations prepare dashboards. We use it to assess the implementation of progress. There is a non-governmental organization that supports kangaroo mother care.

There are quality standards to keep quality health care in our hospital. Based on this, there is an evaluation of the implementation of evidence-based practice of nurses and midwives. Based on the assessment, there is an intervention. Leaders also participate in these activities. The access of material should be addressed by leaders and a training cascade should be provided for nurses and midwives.

I am a unit leader of quality assurance of healthcare and I established a nursing audit committee. I prepare nursing protocols and provide training for nurses. I have not done as important as expected in the case of research studies. However, we use the knowledge from research to identify factors of neonatal mortality rate. We got a reference from the literature that indicated prematurity, sepsis, asphyxia and others are the major causes of neonatal mortality rate. Prematurity is also the major cause of neonatal mortality rate in our hospital.

I and unit section managers have supportive supervision and controls using a checklist during 1 to 5 discussion sessions. There is sharing of ideas peer review sometimes. Peer review is a contract between two nurses and midwives to evaluate the activities of nurses and midwives. The most common tool is 1 to 5 discussions.

1. **How do you think about the implementation of evidence-based practice in your hospital? How do you perceive IEBP?**

The response of the interviewee: **114**

I do not think that nurses and midwives implement evidence-based practice effectively. I am not clear about sources of evidence used for clinical and healthcare decision-making practice during the implementation of evidence-based practice. I know some of the sources of evidence like hospital protocols, national guidelines and standard books. I do not know that research studies and senior nurses and midwives used as evidence for the implementation of evidence-based practice.

1. **How do you think about the importance of the implementation of evidence-based practice? What are important sources of evidence used for the implementation of evidence-based practice?**

The response of the interviewee: **114**

Implementation of evidence-based practice minimizes mistakes and quality healthcare services can be improved.

Our hospital is established recently. Nurses and midwives use hospital protocols to implement evidence-based practice. Some nurses and midwives use seniors to implement evidence-based practice. There is a discussion among nurses and midwives. I know standard books and training manuals are useful for the implementation of evidence-based practice. We do not use research studies for the implementation of evidence-based practice.

1. **What are the reasons that you do not use evidence like research, standard guidelines, hospital protocols, books for your healthcare and clinical decision-making practice?**

The response of the interviewee: **114**

Nurses and midwives do not implement evidence-based practice because of a shortage of materials like lack of library in our hospital, lack of experienced nurses and midwives, lack of good management system and lack of internet access

1. **How the supports for the implementation of evidence-based practice important?**

The response of the interviewee: 114

Evaluation for the implementation of evidence-based practice is difficult.

Matrons are not task holders. However, if there is a shortage of guidelines, they can communicate with the hospital manager and they can avail guidelines to each ward. The chief manager and medical director should have a meeting to discuss the implementation of evidence-based practice with nurses and midwives.

I have not got stakeholders working on the implementation of evidence-based practice. I know that non-governmental organizations, referral hospitals and other governmental organizations are stakeholders. There have not been done on implementation of evidence-based practice. I use my knowledge and skill during clinical decisions. I use the internet when I get it at different cafeterias and smartphones.

I think health professionals, managers and all level leaders should have a commitment to solve the problem of the implementation of evidence-based practice.

I have no contribution to the implementation of evidence-based practice. However, I understand the importance of the implementation of evidence-based practice and I will participate in the implementation of evidence-based practice.

1. **How do you think about the implementation of evidence-based practice in your hospital? How do you perceive IEBP?**

The response of the interviewee: 115

I can provide the best quality healthcare when I read the latest textbooks, standard guidelines and protocols. You see this, it is the implementation of evidence-based practice.

1. **How do you think about the importance of the implementation of evidence-based practice? What are important sources of evidence used for the implementation of evidence-based practice?**

The response of the interviewee: **115**

Implementation of evidence-based practice is the obligation of nurses and midwives to use national guidelines. This improves civil service. We cannot provide quality health service without the implementation of evidence-based practice and we see this as our routine activities.

All health professionals have standard guidelines. EHSTG (Ethiopian health services transformation guidelines) has a question about the implementation of evidence-based practice. We implement evidence-based practice. During the morning session and presentation, we select cases and the basic problem of the hospital to discuss the cases and problem. We measure this by reporting the presentation before the day of discussion to inform all nurses and midwives to read about the cases. We use books privately. However, I do not think that nurses and midwives use books for the implementation of evidence-based practice.

1. **What are the reasons that you do not use evidence like research, standard guidelines, hospital protocols, books for your healthcare and clinical decision-making practice?**

The response of the interviewee: **115**

There are many reasons that we do not implement evidence-based practice. Negligence is one of the causes that is resulted from a lack of our satisfaction. Managerial administration also contribute to nurses and midwives negligence

We have analyzed staff satisfaction. Based on this, some nurses and midwives have good satisfaction. Some nurses and midwives do not have good satisfaction. The other one is lack of internet access. Due to lack of internet access, nurses and midwives have difficulty to get updated evidence.

There is work overload in our hospital. Nurses and midwives cannot use evidence like guidelines, books, research articles and others during working hours. When I come to standard books, there is no library in our hospital to get and read books. There is a shortage of large rooms. In addition to this, there is a lack of knowledge, skills and attitude. Nurses and midwives do not have equal levels of knowledge, skills and attitude. Attitude is internal. There should be compassionate respective care. There are obstacles to provide compassionate respective care. Nurses’ and midwives’ satisfaction hinders compassionate respective care. This is called compassionate respective care fatigue. If there is environmental discomfort, nurses and midwives abuse patients.

1. **How the supports for the implementation of evidence-based practice important?**

The response of the interviewee: 115

There is supportive supervision and control of the implementation of evidence-based practice. For example, the case is presented including care during morning session so that everybody can understand whether nurses and midwives use guidelines and other evidence during patient care.

During the round sessions, we support nurses and midwives by discussing cases in detail. For example, if the case is pneumonia, we explain about pneumonia. We provide the references for reading.

There are not stakeholders working on the implementation of evidence-based practice. There are not sources of evidence distributed by the health institutions. We get guidelines during training. In this case, non-governmental organizations participated in this regard. Government hospitals also support training. The ministry of health and the health bureau do not distribute guidelines. I have not seen the website of the ministry of health and health bureau to get national guidelines and other evidence. We do not have the means to get research articles except the individual endeavors.

We have criteria to measure nurses’ and midwives’ implementation of evidence-based practice. There is EHSTG (Ethiopian hospital service transformation guideline) to measure this. Ethiopian hospital transformation guideline has many national guidelines and hospital protocols. For example, we use standard treatment guidelines and national guidelines.

There is training on updated evidence. Therefore, those nurses and midwives who get the training provide orientation or presentation during the morning session and other meetings and they transfer knowledge and skill for other nurses and midwives.

We also evaluate quality service based on sources of evidence and there is an action plan based on identified gaps. Then ward managers fill the gap for identified problems.

There is a quality unit committee in this hospital. The committee evaluates quality service monthly. The evaluation is based on the ministry of health standards. In addition to this, there is direct observation of nurses and midwives when they perform their procedures and report documents to identify gaps for intervention.

1. **How do you think about the implementation of evidence-based practice in your hospital? How do you perceive IEBP?**

The response of the interviewee: **116**

There is the communication of colleagues about the implementation of evidence-based practice in our hospital. There are orientation and training on the implementation of evidence-based practice during nursing and midwifery meeting. In addition to this, there is orientation about nursing standards and distributions of guidelines to each class.

1. **How do you think about the importance of the implementation of evidence-based practice? What are important sources of evidence used for the implementation of evidence-based practice?**

The response of the interviewee: **116**

There are different nursing standards. The nursing care plan is one of the standards of nurses. There are also other standards like medication administration. These standard guidelines and standard of procedures are available in all workplaces of nurses and midwives.

Nurses and midwives get sources of evidence like research articles and guidelines from the internet. They also use books and their colleagues for the implementation of evidence-based practice .Most nurses and midwives use guidelines for the implementation of evidence-based practice. Few nurses and midwives may use research articles for the implementation of evidence-based practice.

1. **What are the reasons that you do not use evidence like research, standard guidelines, hospital protocols, books for your healthcare and clinical decision-making practice?**

The response of the interviewee: **116**

We do not implement evidence-based practice because of work overload, lack of concentration for sources of evidence, lack of getting evidence, lack of awareness about evidence, shortage of updated guidelines, lack of motivation, lack of skill, shortage of in-service training and we did not develop our skill in skill lab demonstration when we were in colleges and universities.

We do not have a habit of using research articles. There is a lack of perception to use research articles. There is a problem with the attitude to use research articles. There is also a lack of research articles and internet access in our hospital.

Our hospital has no library. However, there are books in the hospital and we use these books. There is a gap in the implementation of evidence-based practice in our hospital. I think nurses and midwives implement evidence-based practice in our hospital.

1. **How the supports for the implementation of evidence-based practice important?**

The response of the interviewee: 116

Generally, there is supportive supervision and control of nurses and midwives to implement evidence-based practice.

There are stakeholders like a quality unit officer in our hospital. In addition to this, the ministry of health, the health bureau, and non-governmental organizations are stakeholders. However, as far as I know, there are not stakeholders that support the implementation of evidence-based practice. I know that I have raised a question about these issues during a staff meeting. The hospital manager is the main responsible body for the implementation of evidence-based practice.

1. **How do think about the implementation of evidence-based practice in your hospital? How do you perceive IEBP?**

The response of the interviewee: **117**

I understand that nurses and midwives should use updated guidelines, textbooks, protocols and research findings. However, some procedures have no guidelines. We use our knowledge that we get from colleges and universities for these procedures in clinical and healthcare decision-making practice. We should also use updated standard books and guidelines.

1. **How do you think about the importance of the implementation of evidence-based practice? What are important sources of evidence used for the implementation of evidence-based practice?**

The response of the interviewee: **117**

There is training for nurses working in the outpatient department. For example, there are sexually transmitted infections, malaria and other training. There is staff communications about updated evidence.

Nurses and midwives perform their professional activities according to their job description. They also participate in management activities. They use national guidelines and standards to develop their knowledge, skills and attitude. There are hospital protocols and policies prepared in the hospital. In addition to this, nurses and midwives get training to develop their knowledge and skills.

Implementation of evidence-based practice is useful for the community. The drug is ordered for the patient according to standard guidelines. We can get guidelines from the internet. We can find getting updated evidence through the internet.

1. **What are the reasons that you do not use evidence like research, standard guidelines, hospital protocols, books for your healthcare and clinical decision-making practice?**

The response of the interviewee: **117**

There are reasons that prevent the implementation of evidence-based practice are lack of internet access, shortage of getting updated guidelines, lack of awareness of nurses and midwives, lack of attention of higher officials or decision-makers, lack of motivation of nurses and midwives, shortage of time, lack of library and lack of attitude.

We cannot use evidence without recognition by the hospital committee. The hospital protocols can be prepared by the committee which has different disciplines.

Our hospital has a human resource problem. The hospital provides services like referral hospitals even though it is a primary hospital.

1. **How the supports for the implementation of evidence-based practice important?**

The response of the interviewee: 117

There is supportive supervision and control of nurses and midwives’ implementation of evidence-based practice.

Some stakeholders support the implementation of evidence-based practice like decision makers, hospital management committees, health bureau, ministry of health, non-governmental organizations and others.

1. **How do you think about the implementation of evidence-based practice in your hospital? How do you perceive IEBP?**

The response of the interviewee: **118**

Implementation of evidence-based practice applying knowledge and skills obtained from the best and current sources of evidence in the healthcare practice.

1. **How do you think about the importance of the implementation of evidence-based practice? What are important sources of evidence used for the implementation of evidence-based practice?**

The response of the interviewee: **118**

Implementation of evidence-based practice is useful for the patient. The patient can get quality health care so that treatment outcome is improved. It is important for patient satisfaction and it minimizes health professional accountability.

Nurses and midwives know the implementation of evidence-based practice. However, we do not implement evidence-based practice. This indicates that knowledge is different from practice. We may not use all sources of evidence to implement evidence-based practice.

First of all, nurses and midwives have got knowledge from their school. In addition to this, they develop their knowledge and skill using guidelines, books, posters and others. We can get newly updated sources of evidence through in-service training. Nurses and midwives get new updated evidence during the morning sessions.

Nurses and midwives get books, guidelines and other sources of evidence from the internet individually. We can use research articles to implement evidence-based practice

1. **What are the reasons that you do not use evidence like research, standard guidelines, hospital protocols, books for your healthcare and clinical decision-making practice?**

The response of the interviewee: **118**

Nurses and midwives do not implement evidence-based practice because of lack of access to evidence, negligence, lack of knowledge, lack of updated evidence, lack of attitude and skill and work overload. For example, we have to provide antenatal care service for 30 pregnant women daily but one midwife provides antenatal care service for around 80 pregnant women daily on average. Therefore, we cannot satisfy the patient and it is impossible to implement evidence-based practice.

We do not use research articles because of lack of access to this evidence and lack of awareness and interest of nurses and midwives. In addition to this, we do not have a library and adequate internet access.

Nurses and midwives are inclined to accept horizontal sharing of evidence instead of vertical sharing of evidence. We do not believe in sharing evidence among colleagues of similar professions. Moreover, our colleague may not know more than I know.

1. **How the supports for the implementation of evidence-based practice important?**

The response of the interviewee: 118

Some stakeholders can support the implementation of evidence-based practice. These stakeholders are matrons, case managers, chief clinical officers, hospital managers, the health bureau, the ministry of health and non-governmental organizations.

I have a plan for morning sessions and seminar sessions to share ideas, knowledge and skills. We planned to have three morning sessions per week. However, we have not had morning sessions and seminar sessions in our hospital.

I may not share evidence and knowledge. However, one may not learn in words. One can learn during practical observation. For example, I do not tell how to secure an intravenous line but one can learn during observation when I secure an intravenous line.

There is updating of sources of evidence through mentoring. However, as far as I know, there are no special activities for the implementation of evidence-based practice. There is weak support of the implementation of evidence-based practice. Non-governmental organizations are interested in family planning and abortion in our hospital.

1. **How do you think about the implementation of evidence-based practice in your hospital? How do you perceive IEBP?**

The response of the interviewee: **119**

First of all, evidence-based practice is the use of hospital protocols, guidelines and training manuals for health care service especially when we give patient care and do procedures. It is a matter of reading books and search for research findings. It is also to get updated information during morning sessions and seminar presentations from experts’ experience.

1. **How do you think about the importance of the implementation of evidence-based practice? What are important sources of evidence used for the implementation of evidence-based practice?**

The response of the interviewee: **119**

I know the evidence such as research articles, guidelines, hospital protocols and others. There is evidence under each case team. I think these sources of evidence are very important for clinical activities in the hospital.

We have guidelines for each ward and case team. Nurses and midwives can read these sources of evidence to use in clinical decisions. However, we do not use research articles for the implementation of evidence-based practice. We use guidelines and books.

1. **What are the reasons that you do not use evidence like research, standard guidelines, hospital protocols, books for your healthcare and clinical decision-making practice?**

The response of the interviewee: **119**

Our hospital has a library and library representative. The library has alphabetically arranged books. Nurses and midwives can borrow books from the library. However, there is limited space in the library and nurses and midwives cannot read books in the library

Nurses and midwives do not implement evidence-based practice because of workload, lack of internet access in each class, limited access to different material and lack of staff motivation. There is a very great burden of workload in our hospital. We have 400 to 500 patients daily. However, it is a primary hospital and it has limited staff members. The hospital-level should be improved to the general hospital.

1. **How the supports for the implementation of evidence-based practice important?**

The response of the interviewee: 119

There is supportive supervision and controls of implementation of evidence-based practice of nurses and midwives. There is an interview of nurses and midwives about hospital protocols and guidelines. In addition to this, there is a chart review. Therefore, we can check the report of the chart with guidelines and hospital protocols.

Higher officials, case managers, zone department, health bureau, ministry of health and non-governmental organizations are stakeholders for the implementation of evidence-based practice.

The health bureau provides us guidelines. There are not non-governmental organizations that support the implementation of evidence-based practice. Previously, there were books provided for this hospital from non-governmental organizations

I participate to prepare hospital protocols. I create awareness of the implementation of evidence-based practice. However, I did nothing about research findings. I place guidelines for each room.

1. **How do you think about the implementation of evidence-based practice in your hospital? How do you perceive IEBP?**

The response of the interviewee: **120**

I think there is the implementation of evidence-based practice in our hospital. For example, nurses and midwives were graduated from universities. We have job descriptions. We do procedures based on job descriptions. For example, I can follow laboring mothers using basic emergency obstetric and newborn care (BEONC) checklist and partograph. There is also immediate postpartum care and newborn care checklist to give care for mother and newborn. I can say this is the implementation of evidence-based practice.

I cannot say nurses and midwives don’t implement evidence-based practice in our hospital. There is a low implementation of evidence-based practice. We use our effort to search sources of evidence from mobile data and there is a discussion about cases among nurses and midwives.

1. **How do you think about the importance of the implementation of evidence-based practice? What are important sources of evidence used for the implementation of evidence-based practice?**

The response of the interviewee: **120**

There haven’t been any sources of evidence provided for nurses and midwives in the hospital. We are hospital workers and we discussed sources of evidence during meetings. For example, there could be postpartum management or essential newborn care case presentation. We learn during a discussion. We get training manuals from training and we place manuals in the ward to be used by nurses and midwives. There are no other means to get sources of evidence. We do not have any guidelines provided by the government.

If our hospital has library and internet access, we would use different sources of evidence for the implementation of evidence-based practice. We can get key performance indicators (KPI) from research articles through searching from the internet.

We cannot get guidelines for the implementation of evidence-based practice. We use our effort to implement evidence-based practice. We download books. I can also get books in the form of soft copies from university teachers. However, we cannot get any other sources of evidence from our hospital.

1. **What are the reasons that you do not use evidence like research, standard guidelines, hospital protocols, books for your healthcare and clinical decision-making practice?**

The response of the interviewee: **120**

The major issue is the lack of access to sources of evidence. We cannot get books in the form of hard and soft copies. There are no guidelines, lack of library, lack of computers and lack of internet access in our hospital.

Work overload is one of the major barriers to the implementation of evidence-based practice. This is very difficult, especially in maternal and child healthcare. You can imagine how you are suffering to get time to interview me. The other barrier is the lack of interest of nurses and midwives. We are interested in updating our level of education. However, there is not education opportunity. Due to this, we are less interested.

We are pushed to shift our profession. Midwives regret to join the midwifery profession. Midwives do not want to update their level of education in midwifery. We have complained about the profession. To tell you the truth, there is complaining of payment and suffering from work overload. I am the head of maternal and child healthcare. I understand our source of the complaint. There is also fear of accountability and lack of uniform healthcare if nurses and midwives use any sources of evidence individually. We cannot use evidence because of this problem.

I asked the hospital to get a computer. We need internet, computer, library and guidelines. However, we haven’t got these.

1. **How the supports for the implementation of evidence-based practice important?**

The response of the interviewee: 120

There is not supportive supervision and control of implementation of evidence-based practice.

The stakeholders are case managers, hospital managers, health bureau and non-governmental organizations. However, we cannot get NGOs.

Although there is a lack of knowledge, skill and attitude, it is easy to fill the gap using senior nurses and midwives. There are Msc nurses and midwives who supervise quality health care services. Our case manager has discussed based on the identified gaps during a staff meeting.

We are the solution for these issues. For example, I have got books with my effort. I cannot get sources of evidence from this hospital. We have to use evidence by our effort. For example, I use my books to manage postpartum hemorrhage. When I forget augmentation and induction protocol, I use my mobile to get the evidence for the implementation of evidence-based practice.

1. **How do you think about the implementation of evidence-based practice in your hospital? How do you perceive IEBP?**

The response of the interviewee: **121**

I am working in all wards and my colleagues too…. I do not expect knowledge and skill at the competency level to use evidence like standard guidelines, books, and research findings. I do not think that we have the knowledge and the skill to perform every procedure using evidence particularly quality research. Some of the nurses and midwives may not be positive to read the evidence. They are negligent in using evidence*.*

1. **How do you think about the importance of the implementation of evidence-based practice? What are important sources of evidence used for the implementation of evidence-based practice?**

The response of the interviewee: **121**

The sources of evidence are hospital protocols, guidelines, training and training manuals, books, research articles and nurses and midwives during sharing of knowledge and skills. Sources of evidence are confirmed to be tested before the use for the implementation of evidence-based practice.

Nurses and midwives use guidelines for the implementation of evidence-based practice. We also use our knowledge that we get from training and education.

We use a study result of researches to implement evidence-based practice. For example, the induction augmentation dose was 2.5 IU previously. This has been proved through a study result that it increases uterine rupture. The dose is decreased to 2 IU because of this reason.

1. **What are the reasons that you do not use evidence like research, standard guidelines, hospital protocols, books for your healthcare and clinical decision-making practice?**

The response of the interviewee: **121**

Nurses and midwives do not implement evidence-based practice mainly because of a shortage of time. There are not adequate nurses and midwives in this hospital. We tried to give services to all patients who have come to this hospital. We do not read books and other sources of evidence rather than covering our work. We do not have time to read books. In addition to this, there is a lack of access to guidelines, research articles and other materials.

Regarding research articles, it should be approved before nurses and midwives use research articles for the implementation of evidence-based practice. We cannot use research articles individually because of fear of accountability. New research results should be approved according to our setup. This should be tested first by seniors of a referral hospital. If it is effective, awareness should be created and it has to be used for the implementation of evidence-based practice. The health bureau should support this activity. Generally, we do not use sources of evidence unless allowed by the health bureau to do so

I am working in all wards and my colleagues too…. I do not expect knowledge and skill at the competency level from all of us. I do not think that we have the knowledge and the skills to perform every procedure. Some nurses and midwives are not positive for the patient. They are angry and negligent to the patients. This is one of the indications of lack of attitude. There is also a lack of in-service training in our hospital.

There is no administrative influence in our hospital. However, it is a rural primary hospital and there is a lack of materials. For example, there is a lack of internet access, books and other access. This should be improved. I was asked to provide my guidelines for the hospital by the managers when I have joined this hospital. I gave my guidelines for the hospital and it is used for postpartum hemorrhage and preeclampsia management.

1. **How the supports for the implementation of evidence-based practice important?**

The response of the interviewee: 121

There is supportive supervision and control of the implementation of evidence-based practice. For example, we use a checklist to confirm postpartum hemorrhage and preeclampsia management are performed based on evidence. I told you that nurses and midwives record procedures based on evidence and this can be identified based on the checklist. Sharing of sources of evidence can be observed during practical procedures and morning sessions.

I know the stakeholders like the health bureau, ministry of health, ward heads, case managers, hospital managers, nurses and midwives, non-governmental organizations. I have not communicated with stakeholders. I have not taken measures for the implementation of evidence-based practice.

I support the implementation of evidence-based practice when I am working on each unit. I have not done anything on research articles and books. I share knowledge and skills during morning sessions and practical case management.

1. **How do you think about the implementation of evidence-based practice in your hospital? How do you perceive IEBP?**

The response of the interviewee: **122**

Newly engaged nurses and midwives after graduation perform procedures similar to students’ attachment performance. They share evidence from senior nurses and midwives working during their attachment. However, they are better for their knowledge and skills sharing. The problem is senior nurses and midwives working in their attachment sites share traditional skills and knowledge for them. The traditional knowledge and skills may not be scientific.

Let me tell you a simple example. Old nurses use swabs before the injection of drugs. Students do this during their clinical attachment. Swabbing is not recommended in infection prevention guidelines. There are also procedures similar to this.

One can see or hear the skills or knowledge during morning sessions and or seminars. One can continue to do the procedures without evidence or he does not have a scientific reason.

There is a new invention in science. It is difficult to say we are implementing evidence-based practice. There are hospital reforms and standards distributed to all hospitals which help to provide uniform healthcare services. The reform approves the presence of procedures guidelines, well-done procedures and it helps to have scientific evidence. There are not research journals and guidelines.

1. **How do you think about the importance of the implementation of evidence-based practice? What are important sources of evidence used for the implementation of evidence-based practice?**

The response of the interviewee: **122**

Textbooks are not accessible in our hospitals. These are available in universities. There is no library in our hospital. Even though there are not books in hard copies, we can avail books in soft copies on desktops to each class. The hospital and professional have the problem of accessing twenty to thirty books on the desktop to be used for the implementation of evidence-based practice.

I know the evidence such as guidelines, hospital protocols, training manuals, standard books, research findings and knowledge and skills shared during morning sessions and seminar presentations from experts. However, we do not use all these sources of evidence for the implementation of evidence-based practice in our hospitals. Most of the time, we use guidelines for the implementation of evidence-based practice. These guidelines are PMTCT, TB, HIV/AIDS and other guidelines. We don't have guidelines for some procedures.

Guidelines are not distributed by the health bureau and ministry of health. However, we can get guidelines from training. Therefore, guidelines should be distributed to health institutions before training after it is prepared by the ministry of health, the world health organization and other responsible bodies.

We use guidelines for the implementation of evidence-based practice. We do not use research articles published from universities and journals. We cannot get these articles. Those who have the interest in searching articles and investigate researches use this evidence. Other nurses and midwives focus on regular work. There are no nurses and midwives who use research articles for the implementation of evidence-based practice.

Most procedures have no guidelines. If you take a prophylaxis antibiotic, there is no guideline. Those who learned in the Black Lion Hospital, they order according to this hospital protocol after their graduation. Some health professionals order prophylaxis antibiotics for 24 hours. Others order a single-dose antibiotic as prophylaxis. Some of them order ampicillin and gentamycin. There are many guidelines similar to this.

Some procedures have training manuals. However, there are no national guidelines. If you take obstetric procedures, there are no national guidelines except selected management of obstetric cases of Addis Ababa University. There are no guidelines for most nurses’ and midwives’ procedures.

1. **What are the reasons that you do not use evidence like research, standard guidelines, hospital protocols, books for your healthcare and clinical decision-making practice?**

The response of the interviewee: **122**

Nurses and midwives use guidelines, books and hospital protocols. However, it is not easy to get books, guidelines, and recent and best research findings. Nurses and midwives do not know the best research findings. They do not have the skill to use it and we need training. We cannot get updated guidelines and manuals for most of the procedures.

Textbooks are not accessible in our hospitals. These are available in universities. There is no library in our hospital. Even though there are no books in hard copies, we can avail books in soft copies on desktops to each class. The hospital and professional have a problem to access twenty to thirty books on the desktop to be used for the implementation of evidence-based practice.

Most procedures have no guidelines. If you take prophylaxis antibiotics, there is no guidelines. Those who learned in the Black Lion Hospital, they order according to this hospital protocol after their graduation. Some health professionals order prophylaxis antibiotics for 24 hours. Others order a single dose antibiotic as prophylaxis. Some of them order ampicillin and gentamycin. There are many guidelines similar to this.

Nurses and midwives do not implement evidence-based practice because of a lack of access to guidelines, journals and other evidence. There is no presentation of updated evidence for health service providers. Ministry of health, health bureau, non-governmental organizations and other partners have to take the responsibility of distribution of guidelines before training of nurses and midwives.

When I say the system, it is morning session and seminar presentation which facilitate the learning process. We can introduce evidence or create awareness for nurses and midwives about sources of evidence. Our morning session is a report presentation. It is a matter of saying 5 caesarean sections, 3 forceps and other reports. It is not based on scientific explanation.

I do not think that lack of motivation and less monthly payment cannot prevent the implementation of evidence-based practice. There is not work overload in our hospital.

I want to think about the workload of general and referral hospitals. There is work overload in these hospitals. However, there are many nurses and midwives in primary hospitals and health centers with less clinical activity. The higher-level hospitals have adequate nurses and midwives but these hospitals serve more than five higher-level hospitals.

Health managers of the ministry of health to hospitals want to participate in politics. Their concern is about to report and it is not about the quality of health services.

Our hospital has no library and computers. There is a gap to place soft copies on desktops and hard copies to each ward. We can buy books for 20,000 birr but we can get two desktops at the same prices and we can have 200 and more standard books on one desktop.

Nurses and midwives think to read only for education. Therefore, they have to read for health care services. Getting knowledge of new evidence should be a habit of nurses and midwives. There should be in-service training.

1. **How the supports for the implementation of evidence-based practice important?**

The response of the interviewee: 122

There is not planned supportive supervision and controls of the implementation of evidence-based practice. However, there are 4 key performance indicators. The report is based on key performance indicators. We try to evaluate the reason for decreasing the performance.

There are stakeholders like heads, quality officers, health bureau, universities, non-governmental organizations and others. I wrote a letter of support for universities to get books. When I got the opportunity, I ask non-governmental organizations to give us guidelines.

My support for the implementation of evidence-based practice of nurses and midwives is not as much as I expected but I did little. I was supporting when I was a quality health assurance officer. I tried to write letters for Bahir Dar University to get books. I tried to place guidelines on each ward.

A Professional association like the midwifery association should create awareness of sources of evidence like guidelines and other sources of evidence.

When there are updated sources of evidence, it is better to present in the hospital. The health bureau should send circulars about the updated evidence. Nurses and midwives have the problem of searching and using sources of evidence. Some of them use their previous knowledge and skills to perform their procedures. There is a problem with attitude and system.

1. **How do you think about the implementation of evidence-based practice in your hospital? How do you perceive IEBP?**

The response of the interviewee: **123**

I think the implementation of evidence-based practice a means to improve quality health services. It is using the best and current sources of knowledge and skills.

1. **How do you think about the importance of the implementation of evidence-based practice? What are important sources of evidence used for the implementation of evidence-based practice?**

The response of the interviewee: **123**

Implementation of evidence-based practice is advantageous for the patient and health service providers. It has great value for quality health service. We perform our activities based on evidence in this hospital. However, the evidence may not be in line with guidelines and other updated sources of evidence. There are not protocols in this hospital.

Sources of evidence are always updated. Some nurses and midwives get these sources of evidence timely and they use the evidence for the implementation of evidence-based practice. Individual activities decide to get updated evidence. Some nurses and midwives use outdated evidence. Implementation of evidence-based practice is useful for the patient, hospital and health service providers.

Most nurses and midwives use sources of evidence that they got from their previous knowledge and skills. Medicine is dynamic because of the intention of newly updated evidence. Some nurses and midwives get training on updated evidence. If one gets the training, he shares the training with the rest of the nurses and midwives. We do not use research articles for the implementation of evidence-based practice. It is not feasible to use articles for the implementation of evidence-based practice.

Unless the ministry of health distributed research findings in the form of guidelines, we do not use it. We can use research findings when we are sure of the harmlessness for our patients. However, I cannot use the sources of evidence individually. The hospital can use the evidence after creating awareness for all health service providers.

Nurses and midwives use guidelines more than books for the implementation of evidence-based practice. I do not think that all nurses and midwives use guidelines for the implementation of evidence-based practice. No one uses books for the implementation of evidence-based practice. Someone may say our hospital has a library. However, nurses and midwives do not know the presence of the library. Even no one knows the service of the library. Some nurses and midwives do not know where the library is.

We use senior nurses and midwives as a source of evidence for the implementation of evidence-based practice informally. We share evidence during routine clinical decisions. We do not have a formal way of sharing evidence among nurses and midwives. When nurses and midwives are transferred from other health institutions to this hospital, there is no orientation to create awareness about sources of evidence.

1. **What are the reasons that you do not use evidence like research, standard guidelines, hospital protocols, books for your healthcare and clinical decision-making practice?**

The response of the interviewee: **123**

Once upon a time, there was a morning session. However, it is interrupted now. We discussed the problems during clinical decisions when we have a meeting. We rise if new sources of evidence are available. There is not any routine meeting to use evidence for the implementation of evidence-based practice. We do not have seminars.

Nurses and midwives do not implement evidence-based practice because of lack of library and reading room, work overload, lack of internet access, lack of computer in nurses and midwifery stations, lack of interest, lack of access to updated evidence and fear of harmfulness of evidence.

Hospitals should find evidence from different sites. There is no this kind of system. I am not quite sure of this communication. I think there is a break between the hospital and health bureau and the ministry of health. There is a need of creating awareness among nurses and midwives.

1. **How the supports for the implementation of evidence-based practice important?**

The response of the interviewee: 123

There is no supportive supervision and controls of implementation of evidence-based practice of nurses and midwives

I know stakeholders like non-governmental organizations, health bureau, and others. I can get a hospital manager and heads. I have also the opportunity to get a non-governmental organization. The hospital manager and heads support the internet access. Non-governmental organizations provide guidelines and manuals for the hospital.

The ministry of health should distribute updated evidence for health centers and hospitals. It is possible to distribute evidence through email, websites and other addresses.

1. **How do you think about the implementation of evidence-based practice in your hospital? How do you perceive IEBP?**

The response of the interviewee: 124

I do have the perception of the implementation of evidence-based practice. However, I do not have a clear idea about the implementation of evidence-based practice.

1. **How do you think about the importance of the implementation of evidence-based practice? What are important sources of evidence used for the implementation of evidence-based practice?**

The response of the interviewee: **124**

Implementation of evidence-based practice is useful for the development of health service providers, improvement of quality health service for the patients and service provision based on ethics. There is a failure of implementation of evidence-based practice. There are different guidelines. However, there is a gap in using these sources of evidence.

Nursing service has great gaps. For example, a nursing care plan should be performed for all patients. The standard recommends that a nursing care plan should be done within 8 hours of patient admission.

There is a national disease classification of nursing guidelines. Nurses diagnose nursing assessment according to disease classification. Nurses do not perform based on this classification.

It is difficult to think that nurses and midwives use books and research articles for the implementation of evidence-based practice. We do not use these sources of evidence. We use guidelines for the implementation of evidence-based practice.

1. **What are the reasons that you do not use evidence like research, standard guidelines, hospital protocols, books for your healthcare and clinical decision-making practice?**

The response of the interviewee: **124**

There is no internet access in nurses’ and doctors’ stations to use evidence. The standard recommends a computer, printer and internet access to each station. However, there are no access to these recommendations. We do not have a functional library. Nurses and midwives may use telemedicine if they get time.

Nurses and midwives do not implement evidence-based practice because of lack of attention, lack of interest to use updated evidence, the problem of skills, lack of satisfaction, lack of motivation, lack of access to the internet, the lack of reading room, work overload, lack of computer, lack of library, lack of skill lab demonstration, resistance to changes and lack of accountability

Nurses and midwives do not participate during morning sessions and seminar presentations. However, we participate during the round. We have a discussion session for special cases. The discussion includes quality health care.

1. **How the supports for the implementation of evidence-based practice important?**

The response of the interviewee: 124

I ask the chief manager, “how to implement evidence-based practice through training and fulfill resources in the hospital?” I did nothing beyond this. I cannot communicate with higher officials and other stakeholders outside this hospital. The chief manager can do this. I understand using guidelines, books, hospital protocols, training manuals and experts’ opinions to improve quality health care.

There is supportive supervision and control of the implementation of evidence-based practice. We observe procedures to check the implementation of evidence-based practice. The quality team also checks healthcare service.

There are stakeholders like quality unit officers, managers, non-governmental organizations and others. Quality unit health officers support the training of nursing care plans. Non-governmental organizations support our hospital on the training in family planning.

1. **How do you think about the implementation of evidence-based practice in your hospital? How do you perceive IEBP?**

The response of the interviewee: **125**

I think nurses and midwives implement evidence-based practice in our hospital. Nurses and midwives implement evidence-based practice through sharing evidence, using guidelines and using other evidence obtained from the internet.

I have not seen any research articles used to implement evidence-based practice. We only give us key performance indicators in this hospital.

I believe that providing clinical decisions based on sources of evidence is vital. There are sources of evidence at the national level and regional levels for different levels of hospitals. When the patient comes to the hospital, he can get health care services according to evidence-based practice.

1. **How do you think about the importance of the implementation of evidence-based practice? What are important sources of evidence used for the implementation of evidence-based practice?**

The response of the interviewee: **125**

Implementation of evidence-based practice is important to update nurses and midwives and share evidence among us.

Nurses and midwives use guidelines for the implementation of evidence-based practice sometimes. We do not use books for the implementation of evidence-based practice. Most of us know the importance of evidence-based practice. Some nurses and midwives do not accept the implementation of evidence-based practice.

1. **What are the reasons that you do not use evidence like research, standard guidelines, hospital protocols, books for your healthcare and clinical decision-making practice?**

The response of the interviewee: **125**

There is a turnover of nurses and midwives in this hospital. We do not adapt to the hospital within a short period. Therefore, this influences the implementation of evidence-based practice.

Nurses and midwives do not implement evidence-based practice because of lack of access like books, lack of internet, lack of guidelines and lack of functional computer, workload, lack of motivation and lack of supportive management.

Managers’ and ward heads’ supportive management system is not good.

When we see incentives, the previous two-year carrier structure is withdrawn. I think there is a lack of access.

Nurses and midwives can use research results for the implementation of evidence-based practice. However, the hospital stakeholders should approve the evidence and create awareness for health workers in the hospital.

1. **How the supports for the implementation of evidence-based practice important?**

The response of the interviewee: 125

I do not think that there is not supportive supervision and control of implementation of evidence-based practice of nurses and midwives.

Some stakeholders support the implementation of evidence-based practice like health workers, managers and ward heads, non-governmental organizations and others. Non-governmental organizations support the gynecology ward. These organizations provide resuscitation material and guidelines. However, there is not any support to use research findings.

**Observation**

**Did the hospital have internet and a library?**

**H101**

There is not any internet and library access in the hospital.

**H102**

The hospital has not any internet and library access.

**H103**

There were not any internet access and library in the hospital.

**H104**

There were not any internet access and library in the hospital.

H105

There was no internet access in the hospital

**H106**

There was internet access in the hospital

**H107**

There were not any internet access and library in the hospital.

**H108**

There were no internet access and library in the hospital

**H109**

There were internet access and a library in the hospital

**H110**

There were not any internet access and library in the hospital.

**H111**

There were not any internet access and library in the hospital.

**H112**

There was internet access in the hospital. However, there was no library

H113

There was internet access in the hospital. However, there were no libraries

H114

There was not any functional library in the hospital. There was internet access in the hospital. But there was no internet in each ward.

**Did nurses and midwives have adequate and comfortable working rooms, and guidelines?**

**H101**

Nurses and midwives have adequate and comfortable working rooms and there are guidelines in most of the wards.

**H102**

Nurses and midwives had no comfortable and adequate working room. More than eight nurses and midwives worked in small rooms which cannot accommodate more than three nurses or midwives in some working units.

The infection prevention technique in the delivery ward was neat and clear. There are desktops in each ward and there were soft copies of guidelines and books on the desktop. However, there were no hard copies in the wards.

**H103**

There was no comfortable and adequate classroom for the staff in labor ward, injection and dressing room, antenatal care unit and outpatient ward.

**H104**

There was no comfortable and adequate classroom for the staff in labor ward, injection and dressing room, antenatal care unit, under-five child health care unit and outpatient ward.

**H105**

During the hospital visit, the observation revealed that most of the procedures did not have guidelines and hospital protocols and there were no books and literature in the ward. Moreover, most of the hospitals had no library, computers, and internet access in the wards. The nurse claimed the nonuse of these resources.

However, there were comfortable and adequate rooms for the staff in labor ward, injection and dressing room, antenatal care unit, under-five child health care unit and outpatient ward.

**H106**

There were a comfortable and adequate rooms for the staff in labor ward, injection and dressing room, antenatal care unit, under-five child health care unit and outpatient ward.

**H107**

There were comfortable and adequate rooms for the staff in labor ward, injection and dressing room, antenatal care unit, under-five child health care unit and outpatient ward

**H108**

There were comfortable and adequate rooms for the staff in all wards like labor ward, injection and dressing room, antenatal care unit, under-five child health care unit and outpatient ward.

**H109**

The hospital had comfortable and adequate rooms for the staff in most wards like labor ward, injection and dressing room, antenatal care unit, under-five child health care unit and outpatient ward. However, there were not comfortable rooms for the staff in the emergency ward.

**H110**

There were comfortable and adequate rooms for the staff in labor ward, injection and dressing room, antenatal care unit, under-five child health care unit and outpatient ward.

**H111**

There were comfortable and adequate rooms for the staff in all wards like labor ward, injection and dressing room, antenatal care unit, under-five child health care unit and outpatient ward.

H112

The hospital had no comfortable and adequate rooms for the staff in labor ward, injection and dressing room, antenatal care unit, under-five child health care unit and outpatient ward.

H113

The hospital had comfortable and adequate rooms for the staff in labor ward, injection and dressing room, antenatal care unit, under-five child health care unit and outpatient ward.

H114

There were comfortable and adequate rooms for the staff in all wards like labor ward, injection and dressing room, antenatal care unit, under-five child health care unit and outpatient ward.

**Did nurses and midwives use guidelines during clinical decisions?**

**H101**

They use guidelines during counseling and advice of the clients and practical procedures. There are infection prevention guidelines and neat infection prevention techniques in the labour ward, injection and dressing room, minor and major operation room.

**H102**

Nurses and midwives had guidelines and they use guidelines for counseling and advice during procedural activities.

**H103**

There were guidelines in each ward and nurses and midwives use the guidelines for counseling and advice of clients. There was an infection prevention guideline in the injection room but the nurses did not use the guideline to implement infection prevention techniques.

**H104**

Nurses and midwives had guidelines and they use guidelines during procedural activities. THE infection prevention technique in the delivery ward was neat and clear. However, they did not use guidelines for counseling and advice.

**H105**

Nurses and midwives had no guidelines and they did not use guidelines during procedural activities. The infection prevention technique was neat and clear in all wards and units. However, they did not use guidelines for counseling and advice.

**H106**

Nurses and midwives had no guidelines and they did not use guidelines during procedural activities and counseling and advice of client. The infection prevention techniques were not neat and clear in all wards and units. Midwives use partograph during laboring mother follow-up appropriately to help them for decision.

**H107**

Nurses and midwives had guidelines. However, they did not use guidelines during procedural activities and counseling and advice of clients. The infection prevention techniques were neat and clear in all wards and units.

**H108**

Nurses and midwives had guidelines and they use guidelines during procedural activities and counseling and advice. However, infection prevention technique in the delivery ward was not neat and clear.

**H109**

Nurses and midwives had no guidelines and they did not use guidelines during procedural activities. The infection prevention technique was neat and clear in all wards and units. However, nurses and midwives did not use guidelines for counseling and advice

**H110**

Nurses and midwives had guidelines. However, they did not use guidelines during procedural activities and counseling and advice of the client. The infection prevention techniques were not neat and clear in all wards and units.

**H111**

Nurses and midwives had guidelines and they use guidelines during procedural activities and counseling and advice. However, they did not use guidelines during procedural activities and infection prevention techniques in the delivery ward, family planning unit and dressing and injection room were not neat and clear.

**H112**

Nurses and midwives had guidelines and they use guidelines for counseling and advice. The infection prevention techniques in the delivery ward, family planning unit and dressing and injection room were neat and clear. However, they did not use guidelines during procedural activities and there was no basic emergency obstetric and newborn care guideline.

**H113**

Nurses and midwives had guidelines and they use guidelines for counseling and advice. The infection prevention techniques in the delivery ward, family planning unit and dressing and injection room were neat and clear. However, they did not use guidelines during procedural activities and there was no injection and infection prevention guideline.

**H114**

Most of the wards and units did not have guidelines and nurses and midwives did not use guidelines for counseling and advice. However, there were EPI and family planning guidelines. The infection prevention techniques in the delivery ward, family planning unit and dressing and injection room were neat and clear. However, they did not use guidelines during procedural activities and there were no injection and infection prevention guidelines.

**Do nurses and midwives fill working documents like partograph based on the standards?**

**H101**

Nurses and midwives documented procedures based on the standards and checklists obtained from evidence like partograph which is important for follow-up of laboring mothers for decision.

**H102**

Midwives use partograph during laboring mother follow-up appropriately to help them for decision. However, most nurses and midwives did not use guidelines for their practical activities. There is no infection prevention guideline in the minor operation room.

H103

Nurses and midwives did not use the available guidelines for the implementation of evidence-based practice in all wards and units. Midwives did not document partograph appropriately. They argued that there was no need to record the second stage of labor events on the partograph.

**H104**

Midwives use partograph during laboring mother follow-up appropriately to help them for decision. There were not any infection prevention and injection guidelines in the minor operation room and injection and dressing room.

**H105**

Midwives use partograph during laboring mother follow-up appropriately to help them for decision

H106

Midwives use partograph during laboring mother follow-up appropriately to help them for decision.

**H107**

Midwives use partograph during laboring mother follow-up appropriately to help them for decision. There were not any infection prevention and injection guidelines in the minor operation room and injection and dressing room.

**H108**

Midwives use partograph during laboring mother follow-up appropriately to help them for decision.

**H109**

Midwives use partograph during laboring mother follow-up appropriately to help them for decision.

**H110**

Midwives use partograph during laboring mother follow-up appropriately to help them for decision.

H111

Midwives use partograph during laboring mother follow-up appropriately to help them for decision.

H113

Midwives did not use partograph during laboring mother follow-up appropriately to help them for decision.

H114

Midwives did use partograph during laboring mother follow-up appropriately to help them for decision.

**Do nurses and midwives perform their competencies without difficulty?**

H101

The observation of practical procedures using checklists like basic emergency obstetric and newborn care indicated that nurses’ and midwives’ competency was not maintained.

H102

The observation of practical procedures using checklists like basic emergency obstetric and newborn care indicated that nurses’ and midwives’ competency was not maintained.

H103

Three procedures were observed using checklists like basic emergency obstetric and newborn care, family planning and injection guidelines checklist. The observation indicated that there were no competent midwives and nurses during the observation of these procedures.

H104

Three procedures were observed using checklists like basic emergency obstetric and newborn care, family planning and infection prevention checklist. The observation indicated that there were no competent midwives and nurses during these procedures.

H105

Three procedures were observed using checklists like basic emergency obstetric and newborn care, family planning and infection prevention checklist. The observation indicated that there were no competent midwives and nurses during these procedures.

H106

Three procedures were observed using checklists like basic emergency obstetric and newborn care, family planning and infection prevention checklist. The observation indicated that there were no competent midwives and nurses during these procedures.

H107

Three procedures were observed using checklists like basic emergency obstetric and newborn care, family planning and infection prevention checklist. The observation indicated that there were no competent midwives and nurses during the observations of these procedures.

H108

Procedures like labour and delivery, implanol insertion, injection and dressing were observed using checklists like basic emergency obstetric and newborn care, family planning, infection prevention and injection and dressing checklist. The observation indicated that there were n0 competent midwives and nurses during the observations of these procedures.

H109

Procedures like labour and delivery, implanol insertion, injection and dressing were observed using checklists like basic emergency obstetric and newborn care, family planning, infection prevention and injection and dressing checklist. The observation indicated that there were competent midwives and nurses during the observations of these procedures.

H110

Procedures like labour and delivery, implanol insertion, injection and dressing were observed using checklists like basic emergency obstetric and newborn care, family planning, infection prevention and injection and dressing checklist. The observation indicated that there were no competent midwives and nurses during the observations of these procedures.

H111

Skilled delivery was attended without the steps of procedures. The skill and getting ready to perform the procedure were the practical challenges.

Procedures like labour and delivery, implanol insertion, injection and dressing were observed using checklists like basic emergency obstetric and newborn care, family planning, infection prevention and injection and dressing checklist. The observation indicated that there were not competent midwives and nurses during the observations of these procedures.

H112

Procedures like labour and delivery, implanol insertion, injection and dressing were observed using checklists like basic emergency obstetric and newborn care, family planning, infection prevention and injection and dressing checklist. The observation indicated that there were competent midwives and nurses during the observations of these procedures.

H113

Procedures like labour and delivery, implanol insertion, injection and dressing were observed using checklists like basic emergency obstetric and newborn care, family planning, infection prevention and injection and dressing checklist. The observation indicated that there were no competent midwives and nurses during the observations of these procedures.

H114

Procedures like labour and delivery, implanol insertion, injection and dressing were observed using checklists like basic emergency obstetric and newborn care, family planning, infection prevention and injection and dressing checklist. The observation indicated that there were no competent midwives and nurses during the observations of these procedures.
