# Supplementary material for: Implementation of evidence-based practice: The experience of nurses and midwives
Source: PLoS One. 2021 Aug 27;16(8):e0256600. doi: 10.1371/journal.pone.0256600 (PMC8396772; doi:10.1371/journal.pone.0256600)
Supplement: S2 File — The interview, FGD and observation guides used in the study were both Amharic and English language. (DOCX) [file pone.0256600.s002.docx]

**Tools used to explore the implementation of evidence-based practice**

**COMPLETE CONSENT FORM OF FGD**

The researcher explained the aim of the study. Moreover, to decide at any time if I do not want to participate. Therefore, I assure you that my interest to participate in this study is truly from my knowledge.

If the client refuses, please check using the X mark in this box

Signature of Person administering consent_______________

Date______________________

Client’s Signature (If agree to be involved)_____________

Date_____________________

Identification number: ______________________________

Data collector: ______________________

Name of the hospital--------------------------------------------

For FGD

Archival # ______________

Site/Location ____________________

Number of participants ________

Date ____/____/___E.C

Time

Start time _______________

End time ______________

Moderator _____________

Note Taker_____________

Recorder ____________

Information from the FGD member

Group__________ (to be filled by the moderator)

Age _______

Give code for each member of FGD-----------------

Facility______________

Ward ____________

Work experience__________year

**Introducing self**

Name, workplace, profession, -------

Purpose

Introduction of participants

Ask Socio-demographic variables, nickname----

Ground Rules

Respect others ideas

Active and free participation

Silent mobile

Avoid side talk

**FGD related Questions for the study**

**Engaging questions**

1. How do you perceive the implementation of Evidence-based Practice in your hospital? How do you understand the implementation of evidence-based practice?

**Probing questions for question number 1**

What do you mean by the perception of implementing evidence-based practice? Do you have any information before? How do you think nurses’/ midwives’ perception of the implementation of evidence-based practice?

1. How do you think about the importance of the implementation of evidence-based practice? What are the sources of evidence used for the implementation of evidence-based practice?

**Probing questions for question number 2**

How do you implement evidence-based practice? What are these sources of evidence? How evidence could be used? How do you get trusted evidence?

1. How do you think your / nurses’ and midwives’/ knowledge, skills, and attitude towards the implementation of evidence-based practice?

**Probing questions for question number 3**

How do you explain the knowledge and skill of nurses and midwives in the implementation of evidence-based practice? Do you have the knowledge and skill to use research, standard guidelines, books and protocols in clinical decision-making practice? How do you elaborate your knowledge and skill to use evidence? What is your experience regarding implementing evidence-based practice in your hospital? How do you share your experience of the implementation of evidence-based practice?

1. Why don’t you implement evidence-based practice?

**Probing questions for question number 4**

What are the barriers to the implementation of evidence-based practice? Which barriers are common in your hospital? How do these barriers affect your clinical and healthcare decision-making practice? How do you overcome these barriers?

1. How the supports for the implementation of evidence-based practice important?

**Probing questions for question number 5**

What are the supports for the implementation of evidence-based practice? Who are the supporters of the implementation of evidence-based practice in your hospital? How do you see the support of your managers’ role to implement evidence-based practice? Why do not managers support the implementation of evidence-based practice? How do you describe the supportive supervision, monitoring and evaluation of the implementation of evidence-based practice in your hospital?

**COMPLETE CONSENT FORM OF INTERVIEW**

The researcher explained the aim of the study. Moreover, to decide at any time if I do not want to participate. Therefore, I assure you that my interest to participate in this study is truly from my knowledge.

If the client refuses, please check using the X mark in this box

Signature of Person administering consent_______________

Date______________________

Client’s Signature_____________

Date_____________________

Identification number: ______________________________

Data collector : ______________________

**Information from the interviewee**

1.Age _______ Position_____________

2.Facility/level______________

3.Ward**____________**

4.Work experience__________year

**Introducing self**

Name, workplace, profession, -------

Purpose of study:

**Interview related questions for the study**

**Engaging questions**

1. How do you think about the implementation of evidence-based practice in your hospital? How do you perceive IEBP?

**Probing questions for question number 1**

What is IEBP? How do you explain your experience of IEBP in your hospital? How do you implement evidence-based practice? What are the sources of evidence used for the implementation of evidence-based practice? What are these sources of evidence? How evidence could be used? How do you get trusted evidence? How do you explain your knowledge and skill to implement evidence-based practice? Do you have the knowledge and skill to use research, standard guidelines, books and protocols in clinical/healthcare practice? How do you elaborate your knowledge and skill to use evidence?

1. How do you think about the importance of the implementation of evidence-based practice? What are important sources of evidence used for the implementation of evidence-based practice?

**Probing questions for question number 2**

Do you think that the implementation of evidence-based practice improves quality health care? Do you think the use of research, standard guidelines and books important for clinical and healthcare decision-making practice? How do you get trusted evidence?

1. What are the reasons that you do not use evidence like research, standard guidelines, hospital protocols, books for your healthcare and clinical decision-making practice?

**Probing questions for question number 3**

What are the barriers to the implementation of evidence-based practice? Which barriers are common in your hospital? How do these barriers affect your clinical and healthcare decision-making practice? How do you overcome these barriers?

1. How the supports for the implementation of evidence-based practice important?

**Probing questions for question number 4**

What are the supports for the implementation of evidence-based practice? Who are the supporters of the implementation of evidence-based practice? How do you see the support of your managers’ role to implement evidence-based practice? Why do not managers support the implementation of evidence-based practice? What is your contribution to support the implementation of evidence-based practice? How do you describe the supportive supervision, monitoring, and evaluation of the implementation of evidence-based practice in your hospital?

**Participatory observation related check-list for the study**

Place a “√” in the box if EBP is implemented and facilities are available satisfactorily, an “**X**” if it is **not** implemented **satisfactorily**, or if not observed.

| Observation checklist | Mark |
| --- | --- |
| 1. Midwives /nurses use guidelines during procedures in clinical and healthcare practice |  |
| 1. Apply infection prevention based on the standards |  |
| 1. Provide counseling about necessary topics based on evidence. |  |
| 1. The hospital has a library. |  |
| 1. The hospital has guidelines, book and research articles |  |
| 1. The hospital has internet access |  |
| 1. The hospital has adequate workrooms for nurses/midwives |  |
| 1. Midwife/nurse fill working documents like partograph based on the standards |  |
| 1. Do midwives/nurses perform their competencies without difficulty? |  |

- **Describe all the observed events during data collection based on the check-list**

**ክፍል ሁለት**

**የተሙዋላ የጥናቱ መጠይቆች የስምምነት ፎርም**

**የጥናቱ ርአስ፡-**

**በአማራ ክልል የመንግስት ሆስፒታሎች ውስጥ በሚሰሩ የነርሶች እና ሚድዋይፎች መረጃን መሰረት ያደረገ ተግባራዊ ኣፈጻጸም እና ተጽኖ የሚያሳድሩ ሁኔታዎች**

እኔ ከዚህ በታች በፍርማ ያረጋገጥኩት በዚህ ጥናት ተሳታፊ ለመሆን ስወስን የጥናቱ አላማዎች፤አሰራር እና ቅድመ ሁኔታዎች በግልጽ በመረዳት እና እንዲሁም ከጥናቱ ተሳታፊነት ፈቃደኝነቴን በማንኛውም ደረጃ የማቋረጥ መብቴን በማረጋገጥ ነው፡፡ በጥናቱ ተሳታፊ መሆኔን በፊርማዬ እያረጋገጥኩ እነዚህ መረጃዎች ሁሉ በሚገባ በምረዳው ቛንቛ የተገለጸልኝ መሆኑን በፊርማዬ አረጋግጣለሁ፡፡

የጥናቱ ተሳታፊ ፊርማ------------------------------------------------

ቀን----------/----------/2012 ዓ.ም

የመረጃ ሰብሳቢ ስም-----------------------------------------

መረጃውን የሰበሰበው ሰው ፊርማ--------------------------------------------------------------------

1. **ቡድን (ኤፍጂ ዲ)ን የተመለከቱ መጠይቆች**

መዝገብl # ______________

ቦታ____________________

የተሳታፊዎች ብዛት በቁጥር ________

ቀን ____/____/___ 

**ሰአት**

ውይይት የተጀመረበት ሰአት _______________

ውይይት የተጠናቀበት ሰአት ______________

የውይይቱ አመቻች _____________

የውይይቱን ማስታወሻ የሚይዝ_____________

ድምፅ ቀጂ ____________

መረጃ ሰብሳቢ ስም-------------------------------------

ቡድን__________ (የሚሞላው በውይይቱ አመቻች ነው)

ለእያንዳንዱ **ኤፍጂ ዲ(FGD)**  አባል የሚስጥር ምልክት ይስጡ-----------------

የሚሰራበት ሆስፒታል______________

ዋርድ ____________

የስራ ልምድ __________አመት

**እራስን ማስተዋወቅI**

ሰም--------------

የስራ ቦታ--------

ሙያ -------

የተሳታፊዎች ትውውቅ

**ሶሽዎ ማህበራዊ ቫሪያብሎችን መጠየቅ**

ቅጥል ስም ---- ---------------እድሜ----------------------ጾታ------------------------

**የውይይቱ ህገ-ደንብ**

የሌሎችን ሀሳብ ማክበር

ንቁ ተሳትፎ ማድረግ

የሞባይል ስልክ ድምፅ ማጥፋት

የጎንዮሽ ወሬ ማቆም

**ጥያቄዎች**

1. መረጃን መሰረት ያደረገ ሙያዊ የተግባር ኣፈጻጸምን በተመለከተ የእርስዎ አመለካከት ወይም ግንዛቤ ምንድነው?

- መረጃን መሰረት ያደረገ ሙያዊ የተግባር ኣፈጻጸምን መረዳት ማለት ምን ማለት ነው? ከዚህ በፊት የሰሙት ነገር አለ? የነርስና ሚድዋፎችን መረጃን መሰረት ያደረገ ሙያዊ የተግባር ኣፈጻጸምን እንዴት ይገነዘቡታል?

2. በህክምና /ክሊኒካል ተግባራዊ ውሳኔ ወቅት መረጃን መሰረት ያደረገ ሙያዊ የተግባር ኣፈጻጸም ምን ያህል አስፈላጊ ነው?

- ለሙያዊ የተግባር ውሳኔ የሚያገለግሉ መረጃዎችን ያውቃሉ፤ እነዚህን መረጃዎች እንዴት እየተጠቀሙባቸው ነው፤ ተአማኒ መረጃዎችን ለመጠቀም እንዴት ለማግኘት ይችላሉ፤ መረጃዎቹ ምን ምን ናቸው? መረጃዎችን ከየት ለማግኘት እንደምንችል ቢጠቅሱልን? መረጃዎችን እንዴት ለመጠቀም ይቻላል ? መረጃዎቹ እርስዎ ይጠቀሙባቸዋል?

3 የእርስዎ የሉሎች ነርስና ሚድዋፎች መረጃን መሰረት አድርጎ ክሊኒካል/ህክምና ተግባርን ለማከናወን እውቀትን፤ ክህሎትንና አመለካከትን በተመለከተ ምን ያስባሉ?

- መረጃን መሰረት አድርጎ የተግባር ውሳኔ መስጠትን በተመለከተ እውቀትንና ክህሎትን እነዴት ይገልጹታል፤፤ እርስዎ መጽሀፍን፤የጥናት መጽሄቶችንና እስታንዳርድ ጋይድላይኖችን በመጠቀም ክሊኒካል ተግባር ውሳኔ ለመወሰን እውቀቱና ክህሎቱ በበቂ ደረጃ አለኝ ብለው ያስባሉ? መረጃዎችን ለመጠቀም የእርስዎን እውቀትና ክህሎት እንዴት ይገልጹታል? መረጃን መሰረት አድርጎ የክሊኒካለ ሙያዊ ውሳኔ ለመወሰን የእርስዎ ልምድ በሆስፒታሎ ምን ይመስላል? የእርስዎን መረጃን መሰረት በማድረግ የተግባር ውሳኔን የመወሰን ልምዶትን እንዴት ያካፍላሉ?

1. መረጃን መሰረት ያደረገ ሙያዊ የተግባርውሳኔን ለምን አይተገብሩም?

- መጽሀፎችን፤ የጥናት ጽሁፎችንና ስታንዳርድጋይድ ላይኖችን ለክሊኒካል ተግባር ውሳኔ ለመጠቀም የሚከለክሉ ነገሮች ምንድናቸው? መረጃዎችን እንዳንጠቀም ከሚከለክሉ ነገሮች በእርስዎ ሆስፒታል በብዛት ያሉት የትኞቹ ናቸው? እነዚህን ከልካይ ነገሮች እንዴት መቁዋቁም ቻሉ?

1. መረጃን መሰረት ያደረገ የክሊኒካለ/ህክምና ተግባር ኣፈጻጸምን ማገዝ ምን ያህል አስፈላጊ ነው?

- መረጃን መሰረት አድርጎ የክሊኒካለ ተግባር ውሳኔን ለመወሰን የሚያግዙ ነገሮች ምንድናቸው? መረጃን መሰረት አድርጎ ሙያን ለመተግበር የቅርብ ሀላፊዎቾን እገዛ እንዴት ይመለከቱታል? የእርስዎ ሀላፊዎች ለምንድነው መረጃን መሰረት ያደረገ ሙያዊ የተግባር ውሳኔን የማይረዱት?

መረጃን መሰረት ያደረገ የክሊኒካለ/ህክምና ተግባር ኣፈጻጸምን ማገዝ ምን ያህል አስፈላጊ ነው? መረጃን መሰረት አድርጎ የክሊኒካለ ተግባር ውሳኔ መወሰንን በተመለከተ በእርስዎ ሆስፒታል ድጋፋዊ ክትትሉን፤ ቁጥጥሩንና ግምገማውን እንዴት ይገልጹታል?

**2.ለጉዳዩ ቀረቤታ ላላቸው ተጠያቂዎች (ኪእንፎርማት)ን የሚመለከቱ መጠይቀች**

**የተጠያቂው መረጃ**

1.እድሜ -------------------ጾታ------------------------

የስራ ሃላፊነት_____________

2.ሆስፒታል ______________

የሆስፒታሉ ደረጃ---------------------

3.ዋርድ**____________**

4.የስራ ልምድ __________አመት

**እራስን ማስተዋወቅ**

ስም--------------

የስራ ቦታ-----------------

ሞያ-------

የትናቱ ኣላማ:

**ጥያቄዎች**

1.መረጃን መሰረት ያደረገ ሙያዊ የተግባር ኣፈጻጸም ለክሊኒካል ተግባራዊ ውሳኔ የመዋል ጠቀሜታውን በእረስዎ ሆስፒታል እንዴት ያስቡታል?

- በመረጃ ላይ የተመሰረተ ሙያዊ የተግባር ውሳኔ ማለት ምን ማለት ነው? የእርስዎን የሙያ አተገባበር ልምድ በሆስፒታሎ እንዴት ይገልጹታል? እርስዎ በሚሰሩበት ሆስፒታል መረጃን መሰረት ያደረገ ሙያዊ የተግባር ኣፈጻጸም እንዴት እየተተገበረ ነው? የተግባር ሙያዎትን ሲተገብሩ የሚጠቀሙባቸው መረጃዎች ምንድናቸው፤፤ መረጃዎችን ይዘርዝሩልኝ? መረጃዎችን እንዴት ይጠቀሙባቸዋል? ተአማኒ የሆኑ መረጃዎችን እንዴት ያገኙዋቸዋል? የጥናት መጽሄቶችን፤ እስታንዳርድ ጋይድላይኖችንና መጽሀፎችን ለሙያ ተግባር ውሳኔ ለማዋል የእርስዎን እውቀት እነዴት ይገልጹታል? እርስዎ መጽሀፎችን፤ እስታንዳርድ ጋይድላይኖችንና የጥናት መጽሄቶችን ለሙያ ተግባር ውሳኔ ለመጠቀም እውቀቱና ክህሎቱ አሎት፤፤ መረጃዎች ለመጠቀም? የእርስዎን እውቀትና ክህሎት ደረጃ ያብራሩልኝ?

1. መረጃ ላይ ተመስርቶ ክሊኒካል ተግባር ውሳኔ መወሰንን ምን ያህል ጠቀሜታ አለው ብለው ያስባሉ?

- መረጃ ላይ ተመስርቶ ክሊኒካል ተግባር ውሳኔ መወሰንን የጤና አገልግሎት ጥራትን ያሻሽላል ብለው ያስባሉ? መጽሀፎችን፤ እስታንዳርድ ጋይድላይኖችንና የጥናት መጽሄቶችን ለክሊኒካል ተግበር ውሳኔ መስጠት ጥሩ ነው ብለው ያስባሉ፤፤ ጥሩ ከሆነ እንዴት?

1. በመረጃ ላይ የተመሰረተ ክሊኒካል ተግባር ውሳኔን ለመተግበር ወይም መጽሀፎችን፤ እስታንዳርድ ጋይድላይኖችንና የጥናት መጽሄቶችን ለመጠቀም የሚከለክሉ ነገሮች ምንድናቸው?

- መረጃ ላይ ተመስርቶ ክሊኒካል ተግባራዊ ውሳኔን ለመተግበር የሚከለክሉ ነገሮች ምንድናቸው? እነዚህ ከልካይ የሆኑ ነገሮች በእርስዎ ክሊኒካል ተግባር ውሳኔ ላይ ምን ያህል ተጽኖ ያደርጋሉ? ይህን ተጽኖ እንዴት ይቁዋቁወሙታል?

1. መረጃ ላይ ተመስርቶ ክሊኒካል ተግባር ውሳኔ መወሰን ላይ እገዛ ማድረግ ጥሩ ነው?

መረጃ ላይ ተመስርቶ ክሊኒካል ተግባር ውሳኔን ለመወሰን የሚያግዙ ነገሮች ምንድናቸው? መረጃ ላይ ተመስርቶ ክሊኒካል ተግባር ውሳኔን ለመወሰን የሚያግዙ ባለድርሻ አካለቶች እነማናቸው? መረጃ ላይ ተመስርቶ ክሊኒካል ተግባር ውሳኔን ለመወሰን በተመለከተ የእርሶን የቅርብ ሀላፊ እገዛ እንዴት ይመለከቱታል? የቅርበ ሀላፊዎ መረጃ ላይ ተመስርቶ ክሊኒካል ተግባር ውሳኔን ለመወሰን እገዛ የማያደርጉት ለምንድነው? መረጃን መሰረት አድርጎ ክሊኒካል ተግባር ውሳኔ መወሰንን በተመለከተ በእርስዎ ሆስፒታል ድጋፋዊ ክትትሉን፤ ቁጥጥሩንና ግምገማውን እንዴት ይገልጹታል?

**3. ተሳትፎ በማድረግና በመመልከት ዳታን/መረጃን መሰብሰቢያ ቸክ ሊስት**

መረጃን መሰረት ያደረገ ተግባር በአጥጋቢነት የሚፈጸም ከሆነ ይህን “√” ምልክት በሳጥን ውስጥ ያመለከቱ ፡፡ መረጃን መሰረት ያደረገ ተግባር በአጥጋቢነት የማይፈጸም ከሆነ ደግሞ ይህን “X” ምልክት በሳጥን ውስጥ ያመለከቱ፡፡

| የመመልከቻ ቸክ ሊስት | የምልክት ሳጥን |
| --- | --- |
| 1. ሆስፒታሉ ቤተ-መጽሀፍ አለው |  |
| 1. ሆስፒታሉ ኢንተርኔት አለው |  |
| 1. ሆስፒታሉ ለነርሶች/ሚደዋይፎች በቂ የሆነ የመስሪያ ክፍል አለው |  |
| 1. ሆስፒታሉ ጋይድላይኖች፤ መጽሀፎች፤የጥናት ጽሁፎችነሌሎችም መረጃውች አሉት |  |
| 1. አስፈላጊ ርእሶች ላይ የምክር አገልግሎት የሚሰጠው መረጃን መሰረት ተደርጎ ነው |  |
| 1. ነርሶች እና ሚድዋይፎች መረጃዎችን ለምሳሌ ጋይድላይንን ለቅደም ተከተል ክንውን በሙያዊ የተግባር ኣፈጻጸም ወቅት ይጠቀሙባቸዋል |  |
| 1. ነርስና /ሚድዋይፍ በስታንዳርድ መሰረት የበሽታ መተላለፍን ይከላከላሉ (infection prevention) |  |
| 1. ነርስዎች/ሚድዋይፎች መረጃዎችን በስታንዳርድ መሰረት ይመዘግባሉ ለምሳሌ ፓርቶግራፍ በአግባቡ ተሞልቷ |  |
| 1. ነርሶችና ሚድዋይፎች ሙያዊ ስራቸውን ምንም ሳይቸገሩ በብቃት ይተገብራሉ |  |

- ከዚህ በተጨማሪ ለጥናቱ ጠቃሚ የሚሉዋቸውን እና ያዩዋቸን ይጻፉዋቸው
